# Mortality and recurrent vascular events after first incident stroke: a 9-year community-based study of 0·5 million Chinese adults

**DOI:** 10.1016/S2214-109X(20)30069-3

**Published:** 2020-03-18

**Authors:** Yiping Chen, Neil Wright, Yu Guo, Iain Turnbull, Christiana Kartsonaki, Ling Yang, Zheng Bian, Pei Pei, Dongxia Pan, Yidan Zhang, Haiqiang Qin, Yilong Wang, Jun Lv, Ming Liu, Zilong Hao, Yongjun Wang, Canqing Yu, Richard Peto, Rory Collins, Liming Li, Robert Clarke, Zhengming Chen, China Kadoorie Biobank Collaborative Group, Yiping Chen, Neil Wright, Yu Guo, Iain Turnbull, Christiana Kartsonaki, Ling Yang, Zheng Bian, Pei Pei, Dongxia Pan, Yidan Zhang, Haiqiang Qin, Yilong Wang, Jun Lv, Ming Liu, Zilong Hao, Yongjun Wang, Canqing Yu, Richard Peto, Rory Collins, Liming Li, Robert Clarke, Zhengming Chen, Junshi Chen, Robin Walters, Daniel Avery, Derrick Bennett, Ruth Boxall, Fiona Bragg, Yumei Chang, Huaidong Du, Simon Gilbert, Alex Hacker, Michael Holmes, Rene Kerosi, Garry Lancaster, Kuang Lin, John McDonnell, Iona Millwood, Qunhua Nie, Paul Ryder, Sam Sansome, Dan Schmidt, Rajani Sohoni, Jenny Wang, Lin Wang, Xiaoming Yang, Xiao Han, Can Hou, Biao Jing, Chao Liu, Zengchang Pang, Ruqin Gao, Shanpeng Li, Shaojie Wang, Yongmei Liu, Ranran Du, Yajing Zang, Liang Cheng, Xiaocao Tian, Hua Zhang, Yaoming Zhai, Feng Ning, Xiaohui Sun, Feifei Li, Silu Lv, Junzheng Wang, Wei Hou, Mingyuan Zeng, Ge Jiang, Xue Zhou, Liqiu Yang, Hui He, Bo Yu, Yanjie Li, Qinai Xu, Quan Kang, Ziyan Guo, Dan Wang, Ximin Hu, Hongmei Wang, Jinyan Chen, Yan Fu, Zhenwang Fu, Xiaohuan Wang, Min Weng, Zhendong Guo, Shukuan Wu, Yilei Li, Huimei Li, Zhifang Fu, Ming Wu, Yonglin Zhou, Jinyi Zhou, Ran Tao, Jie Yang, Jian Su, Fang Liu, Jun Zhang, Yihe Hu, Yan Lu, Liangcai Ma, Aiyu Tang, Shuo Zhang, Jianrong Jin, Jingchao Liu, Zhenzhu Tang, Naying Chen, Ying Huang, Mingqiang Li, Jinhuai Meng, Rong Pan, Qilian Jiang, Jian Lan, Yun Liu, Liuping Wei, Ningyu Chen, Ping Wang, Fanwen Meng, Yulu Qin, Sisi Wang, Xianping Wu, Ningmei Zhang, Xiaofang Chen, Weiwei Zhou, Guojin Luo, Jianguo Li, Xunfu Zhong, Jiaqiu Liu, Qiang Sun, Pengfei Ge, Xiaolan Ren, Caixia Dong, Hui Zhang, Enke Mao, Xiaoping Wang, Tao Wang, Xi Zhang, Ding Zhang, Gang Zhou, Shixian Feng, Liang Chang, Lei Fan, Yulian Gao, Tianyou He, Huarong Sun, Pan He, Chen Hu, Xukui Zhang, Huifang Wu, Min Yu, Ruying Hu, Hao Wang, Yijian Qian, Chunmei Wang, Kaixu Xie, Lingli Chen, Zhongxi Fu, Qiaohua Xu, Xin Xu, Hao Zhang, Huajun Long, Xianzhi Li, Libo Zhang, Zhe Qiu

**Affiliations:** a Medical Research Council Population Health Research Unit, Oxford, UK; b Clinical Trial Service Unit and Epidemiological Studies Unit, Nuffield Department of Population Health, University of Oxford, Oxford, UK; c Chinese Academy of Medical Sciences, Beijing, China; d Non-Communicable Diseases Prevention and Control Department, Tongxiang Centre for Disease Control, Zhejiang, China; e Beijing Tiantan Hospital of Capital Medical University, Beijing, China; f Department of Epidemiology and Biostatistics, School of Public Health, Peking University Health Science Centre, Beijing, China; g Stroke Clinical Research Unit, Department of Neurology, West China Hospital, Sichuan University, Chengdu, China

## Abstract

**Background:**

Stroke is a leading cause of death and disability worldwide. Despite considerable improvements in diagnosis and treatment, little is known about the short-term and long-term prognosis after a first stroke in low-income and middle-income countries, including China. We aimed to assess the short-term and long-term risk of recurrent stroke and mortality after a first stroke for each of the major pathological stroke types.

**Methods:**

This population-based cohort study included adults aged 35–74 years without disability who were recruited to the China Kadoorie Biobank (CKB). A baseline survey was conducted in ten geographical areas (five urban, five rural) in China, and participants had clinical measurements recorded. Participants were followed up by monitoring death registries and by electronic linkage to health registries and health insurance claims databases, with follow-up until Jan 1, 2017. Participants were excluded from analyses if they had a previous history of stroke, transient ischaemic attack, or ischaemic heart disease at baseline. All incidences of fatal and non-fatal stroke during the study period were recorded by type (ischaemic stroke, intracerebral haemorrhage, subarachnoid haemorrhage, and unspecified type). Primary outcome measures were 28-day mortality, recurrent stroke, major vascular events (recurrent stroke, myocardial infarction, or vascular death), vascular mortality, and all-cause mortality.

**Findings:**

Of 512 715 individuals in the CKB, 489 586 participants without previous ischaemic heart disease and stroke at recruitment were included, of whom 45 732 (42 073 [92%] confirmed by brain imaging) had a stroke during the study period. The mean age was 59·3 years (SD 9·8) for participants who had a stroke (54% women) and 50·8 years (10·3) for participants with no stroke (60% women). 36 588 (80%) of the incident cases of stroke were ischaemic stroke, 7440 (16%) were intracerebral haemorrhage, 702 (2%) were subarachnoid haemorrhage, and 1002 (2%) were an unspecified stroke type. 28-day mortality was 3% (95% CI 3–4) for ischaemic stroke, 47% (46–48)for intracerebral haemorrhage, 19% (17–22; 52% for rural areas and 32% for urban areas) subarachnoid haemorrhage, and 24% (22–27) for unspecified stroke. Among participants who survived stroke at 28 days, 41% (41–42) had recurrent stroke at 5 years (ischaemic stroke 41% [41–42], intracerebral haemorrhage 44% [42–46], subarachnoid haemorrhage 22% [18–27], unspecified stroke type 40% [35–44]) and mortality at 5 years was 17% ([17–18] ischaemic stroke 16% [15–16], intracerebral haemorrhage 28% [26–29], subarachnoid haemorrhage 16% [12–20], unspecified stroke type 15% [12–19]). After a first ischaemic stroke, 91% of recurrent strokes were also ischaemic stroke; after an intracerebral haemorrhage, 56% of recurrent strokes were intracerebral haemorrhage, and 41% of recurrent strokes were ischaemic stroke.

**Interpretation:**

After a first stroke, the risk of recurrence or death within 5 years was high among this population of Chinese adults. Urgent improvements to secondary prevention of stroke in China are needed to reduce these risks.

**Funding:**

Wellcome Trust, Medical Research Council, British Heart Foundation, Cancer Research UK, Kadoorie Charitable Foundation, Chinese Ministry of Science and Technology, National Natural Science Foundation of China.

**Copyright:**

© 2020 The Author(s). Published by Elsevier Ltd. This is an Open Access article under the CC BY 4.0 license.

## Introduction

Stroke is a leading cause of death and permanent disability worldwide. Around three-quarters of the global burden of stroke deaths (approximately 6·5 million per year) and associated disability-adjusted life years (113 million) now occur in low-income and middle-income countries, including China.^1^ In recent decades, the incidence and mortality rates of first stroke cases have declined progressively in high-income countries, but have not changed or have increased in many low-income and middle-income countries.^1^ Stroke is the leading cause of death and disability in China, accounting for more than 3 million new cases and around 2 million deaths in 2013,^2^, ^3^ and a higher proportion of strokes are intracerebral haemorrhages in the Chinese population than in western populations.^2^, ^3^

Previous large, nationwide studies in China have provided reliable estimates for stroke prevalence,^4^ incidence,^3^ and mortality,^1^ and have assessed the role of major risk factors for stroke.^5^, ^6^ However, there is little reliable contemporary evidence in China (and many other low-income and middle-income countries) about the case-fatality rate or recurrent risk after first stroke of different types. Previous such studies have been constrained by small sample sizes,^7^, ^8^ a focus on particular stroke types or minor cases,^7^, ^8^, ^9^ short duration of follow-up,^3^ or restriction to hospital-based rather than community-based settings.^10^ As in most high-income countries, considerable improvements have been made in the diagnosis of stroke (ie, widespread use of brain imaging), the acute treatment of stroke (eg, thrombolysis and antiplatelet therapy), and long-term secondary prevention (eg, blood-pressure lowering, antiplatelet and lipid-lowering therapy) in the past few decades in China.^11^ However, considerable uncertainty persists about whether such advances have resulted in any substantial improvement in both the short-term and long-term prognosis after first stroke in the general population. Reliable contemporary evidence on the natural history of stroke types in low-income and middle-income countries is needed to guide the delivery of appropriate services for the treatment and prevention of stroke.

**Research in context**

**Evidence before this study**

We searched PubMed from inception to Sept 1, 2019, for studies that had investigated the recurrence or mortality after stroke and major stroke types, using the search terms (“stroke” or “Ischemic stroke” or “haemorrhagic stroke” or “Chinese” and “recurrence” or “mortality or fatality”) in articles published in English. In a national representative survey on stroke incidence and mortality, recurrence was not reported and patients were only followed up for 1 year. Although considerable improvements have been made in the diagnosis and treatment of stroke, substantial uncertainty persists about whether such advances have resulted in any appreciable improvements in prognosis after a first stroke in China.

**Added value of this study**

In this prospective study of adults recruited into the China Kadoorie Biobank during 2004–08, 489 586 participants without previous cardiovascular disease events were followed up for 9 years to record cases of first incident and recurrent stroke and cause-specific mortality. Risks of death and recurrent stroke were estimated separately by stroke type. 45 732 participants had a first stroke, including 36 588 (80%) individuals who had an ischaemic stroke, 7440 (16%) who had an intracerebral haemorrhage, 702 (2%) who had a subarachnoid haemorrhage, and 1002 (2%) who had an unspecified stroke type. The prognosis after a first stroke was poor, with 28-day mortality of 3% after ischaemic stroke, 47% after cerebral haemorrhage, 19% after subarachnoid haemorrhage, and 24% after unspecified stroke, with higher 28-day mortality in rural than urban areas, particularly for intracerebral haemorrhage (52% *vs* 32%). Among stroke survivors at 28 days, 41% had recurrent stroke and 17% died by 5 years after first stroke.

**Implications of all the available evidence**

Despite progress in the treatments for stroke worldwide, the short-term and long-term prognosis following stroke is still poor in middle-income countries such as China, highlighting the urgent need for further improvements in secondary prevention of stroke in these countries.

We present relevant findings from a 9-year follow-up of a population-based study of 0·5 million adults from ten diverse geographical areas in China. The aims of this study were to assess the 28-day case-fatality rates after first stroke for the major pathological stroke types; to estimate the short-term and long-term risks of recurrent stroke and mortality after a first stroke event by stroke type; and to investigate the pathological types of recurrent stroke after a first stroke of different types.

## Methods

### Study design and participants

Details of the design and methods used in the China Kadoorie Biobank (CKB) have been previously reported.^12^, ^13^ In brief, the 2004–08 baseline survey was conducted in ten geographical areas (five urban, five rural) of China, chosen from China's nationally representative Disease Surveillance Points. These ten areas were chosen to maximise regional and social diversity, differences in risk exposures, and differences in burden of major diseases, including stroke. In each area, all permanent residents aged 35–74 years without disability were invited to participate. 512 715 individuals (including a small number of individuals outside the target age range) agreed to participate in the study.

Trained health workers administered laptop-based questionnaires at local study clinics, which collected detailed information on sociodemographic status, smoking, alcohol consumption, diet, physical activity, and medical history. All participants also had several clinical measurements recorded, including height, weight, waist and hip circumference, lung function, blood pressure, and heart rate, and had a blood sample collected for long-term storage. Previous international, national and local ethics approval was obtained and all participants provided written informed consent to participate in the study and for their health status to be monitored by linkage with their electronic health records.

### Procedures and outcomes

After the baseline survey, the vital status of participants was monitored regularly through Disease Surveillance Point death registries, supplemented by annual checks with local residential records and active confirmation by contacting local street committees or village administrators.^13^ The causes of death, usually available from official death certificates, were supplemented by available medical records. In the small proportion of deaths (<5%) occurring without recent medical attention, standard procedures (verbal autopsy) were used to determine the probable causes of death based on symptoms and signs provided by informants (usually family members).^14^

All hospitalised cases of stroke (and other diseases) were identified by electronic linkage, via a unique personal identification number, to established registries of major diseases (ie, stroke, ischaemic heart disease, cancer, and diabetes) and to local health insurance claims databases for any hospital admissions, which covered more than 97% of study participants (these procedures also yielded a few additional deaths that had not been identified through death registries). To minimise bias due to loss to follow-up, any uninsured participants were also actively followed up by annual home visits in addition to annual checking of death and disease registries for any hospital admissions with stroke and other diseases and deaths in such participants.

All fatal and non-fatal stroke cases reported by different sources were coded by trained medical staff, who were blinded to other personal information, using the International Classification of Diseases 10th revision (ICD-10), with further checking and review done centrally by a clinical research fellow. Any hospital-reported cases of first stroke also underwent separate clinical adjudication, involving retrieval and review of original medical records and brain imaging reports by clinical specialists in China using a bespoke web-based system.

### First incident and recurrent strokes

For first incident stroke, the major pathological types examined were ischaemic stroke (ICD-10 code I63), including lacunar infarction and non-lacunar infarction; intracerebral haemorrhage (I61); subarachnoid haemorrhage (I60); and unspecified stroke (I64). Ischaemic stroke was defined as a focal neurological dysfunction lasting for more than 24 h with or without neuroimaging evidence of a cerebral infarct. Lacunar infarction was defined as stroke with neuroimaging evidence of an infarct in the brain of less than 1·5 cm in diameter on CT or MRI reports, with or without focal neurological deficits, including lacunar syndrome. Non-lacunar infarction was any other type of ischaemic stroke, excluding lacunar infarction. Intracerebral haemorrhage was defined as neurological dysfunction caused by haemorrhage into the brain parenchyma or the ventricular system, excluding those induced by injury, with or without neuroimaging evidence of brain haemorrhage. Subarachnoid haemorrhage was defined as neurological dysfunction considered to be caused by haemorrhage into the subarachnoid space, excluding those induced by injury, with or without relevant neuroimaging evidence. 92% of the reported first stroke cases had their diagnosis confirmed by brain imaging (CT or MRI). Radiological reports (but not the brain imaging) of individuals with non-fatal stroke were reviewed by Chinese neurologists online.

The primary outcome measures were 28-day mortality, recurrent stroke, major vascular events, vascular mortality, and all-cause mortality. Recurrent stroke was defined as any hospitalised stroke events (ICD-10 I60, I61, I63, I64) occurring more than 24 h after the onset of a known first stroke. Major vascular events were defined as recurrent stroke, myocardial infarction (I21), or vascular death (I00–99), whichever was reported first.

### Statistical analysis

The present analysis included all participants who had a stroke between enrolment and Jan 1, 2017, without a previous history of stroke or transient ischaemic attack, or ischaemic heart disease at baseline. The incidences of first stroke were standardised by sex, area, and age group to the overall CKB population for each decade of age at the time of diagnosis, excluding those with previous ischaemic heart disease or a previous stroke or transient ischaemic attack and are presented as incidence per 100 000 person-years. 28-day mortality was calculated as the proportion of participants who died within 28 days after a first incident stroke.

Cumulative mortality after first incident stroke was calculated as one minus Kaplan-Meier survival probability. The cumulative event rates (95% CIs) for recurrent stroke and other cardiovascular disease outcomes were calculated by the cumulative incidence function, treating death from any cause as a competing risk. The cumulative incidence of competing risks was estimated using the cmprsk package in R and the corresponding 95% CIs were based on the log-minus-log transformation.^15^, ^16^ The event rates were estimated for all participants who had a first incident stroke, and separately by stroke type. Event rates were also calculated separately by sex and area (urban or rural). In analyses of multiple events (vascular or non-vascular deaths; recurrent strokes classified by type; myocardial infarction, angina, or other cardiac diseases), different event types were treated as competing risks in the analyses. Unless otherwise specified, recurrent cases of stroke, major vascular events, or deaths were restricted to those occurring more than 28 days after a first stroke event. All analyses were done using R 3.5.0.

### Role of the funding source

The funders of the study had no role in study design, data collection, data analysis, data interpretation, or writing of the report. YC, RCl, and ZC had full access to all the data in the study and had final responsibility for the decision to submit for publication.

## Results

By Jan 1, 2017 (the censoring date for the present analyses), 44 066 (8·6%) participants had died and 4751 (<1%) were lost to follow-up, mostly because participants moved out of the study area.

Of 512 715 individuals in the CKB, 23 129 had a previous history of stroke, transient ischaemic attack, or ischaemic heart disease at baseline and were excluded from these analyses. 489 586 participants were included, of whom 45 732 had a stroke during the study period and 443 854 had no stroke (table 1).

**Table 1:** Baseline characteristics of participants by type of stroke

		**Stroke type**				**Any stroke (n=45 732)**	**No stroke (n=443 854)**
		IS (n=36 588)	ICH (n=7440)	SAH (n=702)	Unspecified (n=1002)		
Age group, years							
	30–39	974 (2·7%)	285 (3·8%)	52 (7·4%)	36 (3·6%)	1347 (2·9%)	76 013 (17·1%)
	40–49	5662 (15·5%)	1122 (15·1%)	148 (21·1%)	166 (16·6%)	7098 (15·5%)	143 628 (32·4%)
	50–59	12 096 (33·1%)	2202 (29·6%)	259 (36·9%)	282 (28·1%)	14 839 (32·4%)	135 833 (30·6%)
	60–69	12 122 (33·1%)	2467 (33·2%)	172 (24·5%)	323 (32·2%)	15 084 (33·0%)	67 199 (15·1%)
	70–79	5734 (15·7%)	1364 (18·3%)	71 (10·1%)	195 (19·5%)	7364 (16·1%)	21 181 (4·8%)
Age, years		59·3 (9·6)	59·6 (10·2)	56 (10·1)	59·6 (10·4)	59·3 (9·8)	50·8 (10·3)
Sex							
	Men	16 380 (44·8%)	3913 (52·6%)	265 (37·7%)	455 (45·4%)	21 013 (45·9%)	179 122 (40·4%)
	Women	20 208 (55·2%)	3527 (47·4%)	437 (62·3%)	547 (54·6%)	24 719 (54·1%)	264 732 (59·6%)
Area							
	Rural	17 606 (48·1%)	5618 (75·5%)	447 (63·7%)	530 (52·9%)	24 201 (52·9%)	253 985 (57·2%)
	Urban	18 982 (51·9%)	1822 (24·5%)	255 (36·3%)	472 (47·1%)	21 531 (47·1%)	189 869 (42·8%)
Highest education							
	Primary school or no formal education	20 079 (54·9%)	5317 (71·5%)	433 (61·7%)	649 (64·8%)	26 478 (57·9%)	222 431 (50·1%)
	Middle school or high school	13 938 (38·1%)	1931 (26·0%)	244 (34·8%)	317 (31·6%)	16 430 (35·9%)	196 457 (44·3%)
	College or university	2571 (7·0%)	192 (2·6%)	25 (3·6%)	36 (3·6%)	2824 (6·2%)	24 966 (5·6%)
Annual household income, ¥							
	<9999	11 301 (30·9%)	3335 (44·8%)	209 (29·8%)	377 (37·6%)	15 222 (33·3%)	123 610 (27·8%)
	10 000–19 999	11 822 (32·3%)	2095 (28·2%)	224 (31·9%)	280 (27·9%)	14 421 (31·5%)	126 910 (28·6%)
	20 000–34 999	8000 (21·9%)	1291 (17·4%)	159 (22·6%)	199 (19·9%)	9649 (21·1%)	111 274 (25·1%)
	≥35 000	5465 (14·9%)	719 (9·7%)	110 (15·7%)	146 (14·6%)	6440 (14·1%)	82 060 (18·5%)
Current smoker							
	Men	9272 (56·6%)	2414 (61·7%)	160 (60·4%)	254 (55·8%)	12 100 (57·6%)	111 930 (62·5%)
	Women	802 (4·0%)	133 (3·8%)	23 (5·3%)	20 (3·7%)	978 (4·0%)	5642 (2·1%)
Current alcohol drinker							
	Men	11 915 (72·7%)	2525 (64·5%)	182 (68·7%)	305 (67·0%)	14 927 (71·0%)	138 459 (77·3%)
	Women	7596 (37·6%)	982 (27·8%)	165 (37·8%)	175 (32·0%)	8918 (36·1%)	95 510 (36·1%)
Regular dietary intake*							
	Meat	28 811 (78·7%)	5427 (72·9%)	582 (82·9%)	793 (79·1%)	35 613 (77·9%)	369 906 (83·3%)
	Fish	15 636 (42·7%)	2427 (32·6%)	311 (44·3%)	438 (43·7%)	18 812 (41·1%)	209 947 (47·3%)
	Dairy	9029 (24·7%)	859 (11·5%)	129 (18·4%)	181 (18·1%)	10 198 (22·3%)	86 145 (19·4%)
	Fruit	20 468 (55·9%)	3295 (44·3%)	415 (59·1%)	522 (52·1%)	24 700 (54·0%)	266 159 (60·0%)
Prevalent disease†							
	Diabetes	2583 (7·1%)	310 (4·2%)	20 (2·8%)	71 (7·1%)	2984 (6·5%)	10 329 (2·3%)
	Hypertension	7960 (21·8%)	1844 (24·8%)	117 (16·7%)	246 (24·6%)	10 167 (22·2%)	38 392 (8·6%)
Systolic blood pressure, mm Hg		141·5 (24·2)	151·2 (26·6)	138·3 (22·6)	143 (25·7)	143 (24·9)	129·3 (20·2)
Body-mass index (kg/m^2^)		24·3 (3·5)	23·3 (3·6)	23·7 (3·5)	23·8 (3·6)	24·1 (3·5)	23·5 (3·3)
Random plasma glucose, mmol/L		6·7 (3·2)	6·5 (3·1)	6·2 (2·7)	6·6 (3·3)	6·7 (3·2)	6·0 (2·2)

Data are mean (SD) or n (%). 23 129 participants with self-reported previous ischaemic heart disease, stroke, or transient ischaemic attack at baseline were excluded. IS=ischaemic stroke. ICH=intracerebral haemorrhage. SAH=subarachnoid haemorrhage.

* 1–3 days per week or more often.

† Self-reported at baseline survey.

The mean age at baseline was 59·3 years (SD 9·8) for participants who had a stroke during the study period and 50·8 years (10·3) for participants who had no stroke. 54·1% with stroke and 59·6% without stroke were women, and 47·1% with stroke and 42·8% without stroke resided in urban areas (table 1). During 4·72 million person-years of follow-up, 45 732 participants had a first incident stroke, including 36 588 (80%) ischaemic strokes, 7440 (16%) intracerebral haemorrhages, 702 (2%) subarachnoid haemorrhages, and 1002 (2%) unspecified stroke types (table 1). 42 073 (92%) of 45 732 stroke diagnoses were confirmed by brain imaging using CT or MRI.

The standardised event rates were 778 (95% CI 107–113) per 100 000 person-years for ischaemic stroke (110 [107–113] lacunar infarction, 668 [661–676] non-lacunar infarction), 156 (153–160) for intracerebral haemorrhage, 15 (14–16) for subarachnoid haemorrhage, and 21 (20–22) for unspecified stroke types (appendix p 4). The event rate of ischaemic stroke was higher in those living in urban areas rather than rural areas (appendix p 4). In contrast, the event rate of intracerebral haemorrhage were higher in those living in rural areas rather than urban areas (appendix p 4).

Compared with those who had no stroke, individuals who developed a first stroke during the study period tended to be older, male, to reside in urban areas, and to have lower levels of education and household income at baseline (table 1). Compared with ischaemic stroke, individuals who had intracerebral haemorrhage were more likely to be men, to live in rural areas, and to have lower levels of education and income. Individuals who had intracerebral haemorrhage also tended not to regularly consume meat, fish, fruit, and dairy products, and tended to be current smokers and have higher mean systolic blood pressure than individuals who had ischaemic stroke. However, the two groups had a similar mean body-mass index and random plasma glucose concentration. The baseline characteristics of individuals who had subarachnoid haemorrhage were generally similar to those of individuals who had intracerebral haemorrhage, and those of individuals who had unspecified stroke were similar to those of individuals who had ischaemic stroke (table 1).

**Figure 1:**
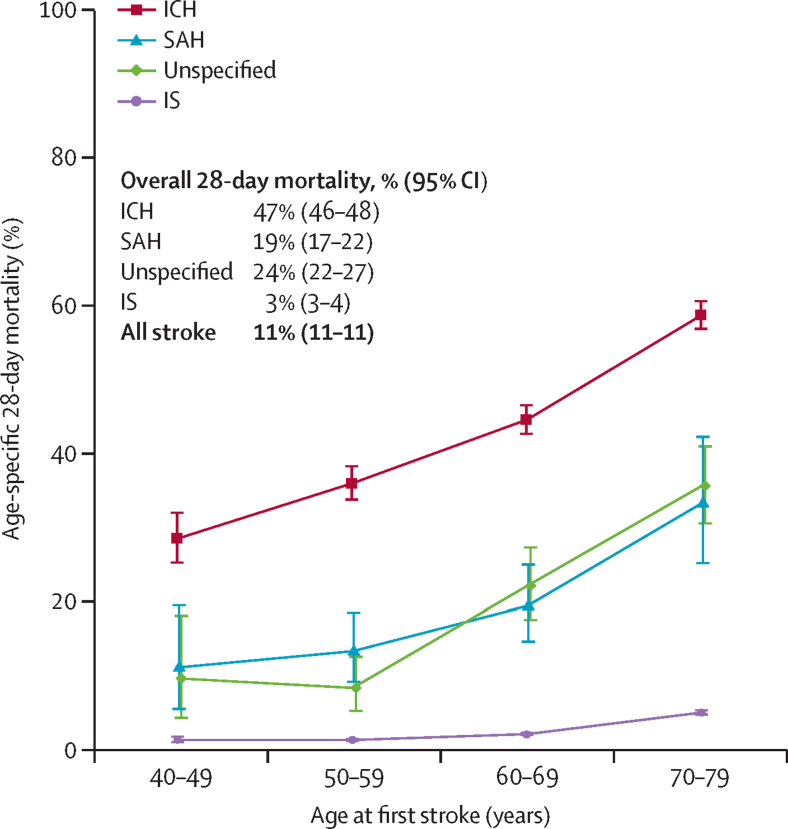
Age-specific 28-day mortality after a first stroke of different types Mortality was calculated as the proportion of participants dying from any cause within 28 days after a first stroke. Whiskers indicate 95% CIs. IS=ischaemic stroke. ICH=intracerebral haemorrhagic stroke. SAH=subarachnoid haemorrhage.

28-day mortality of individuals with first stroke was 11% (95% CI 11–11; figure 1), with higher age-specific mortality in men than women (13% [12–13] *vs* 10% [9–10]; appendix p 13), and in rural areas than urban areas (16% [15–16] *vs* 6% [6–6]; appendix p 13). Mortality increased with age, particularly for intracerebral haemorrhage (figure 1). 28-day mortality was highest for intracerebral haemorrhage (47% [46–48]) and lowest for ischaemic stroke (3% [3–4]). For ischaemic stroke, 28-day mortality was higher for non-lacunar infarction than for lacunar infarction (4% [3–4] vs 0·4% [0·2–0·6]; data not shown).

**Figure 2:**
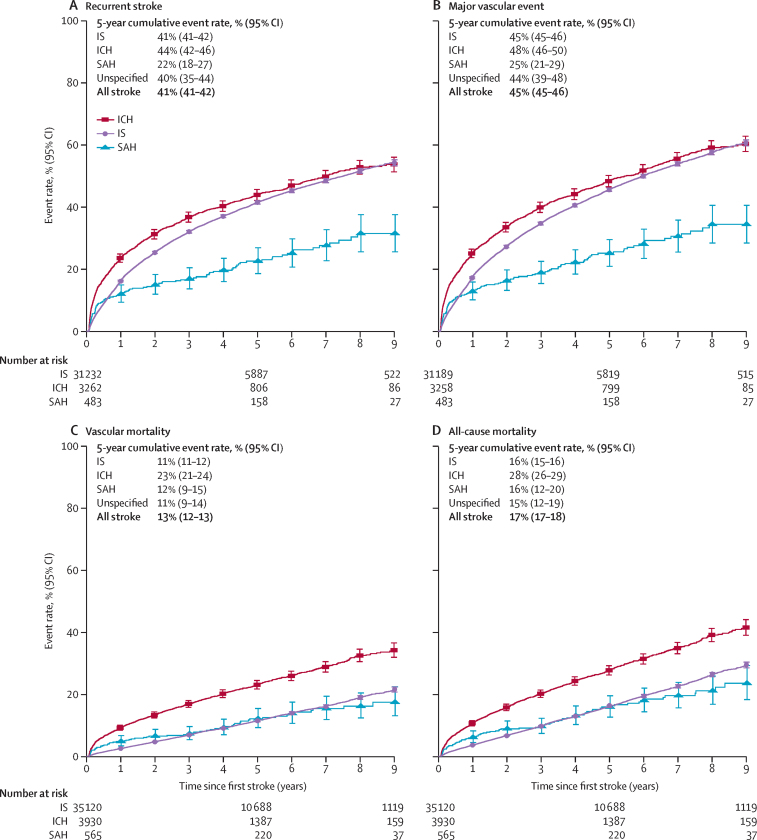
Estimated cumulative event rates of recurrent stroke, major vascular events, vascular mortality, and all-cause mortality from 28 days after first stroke of different types Plotted lines indicate the cumulative incidence, starting at the date of first stroke. Whiskers indicate 95% CIs. Deaths from any cause were treated as competing risks. Participants experiencing an event or death within 28 days from first stroke were excluded. IS=ischaemic stroke. ICH=intracerebral haemorrhagic stroke. SAH=subarachnoid haemorrhage.

**Figure 3:**
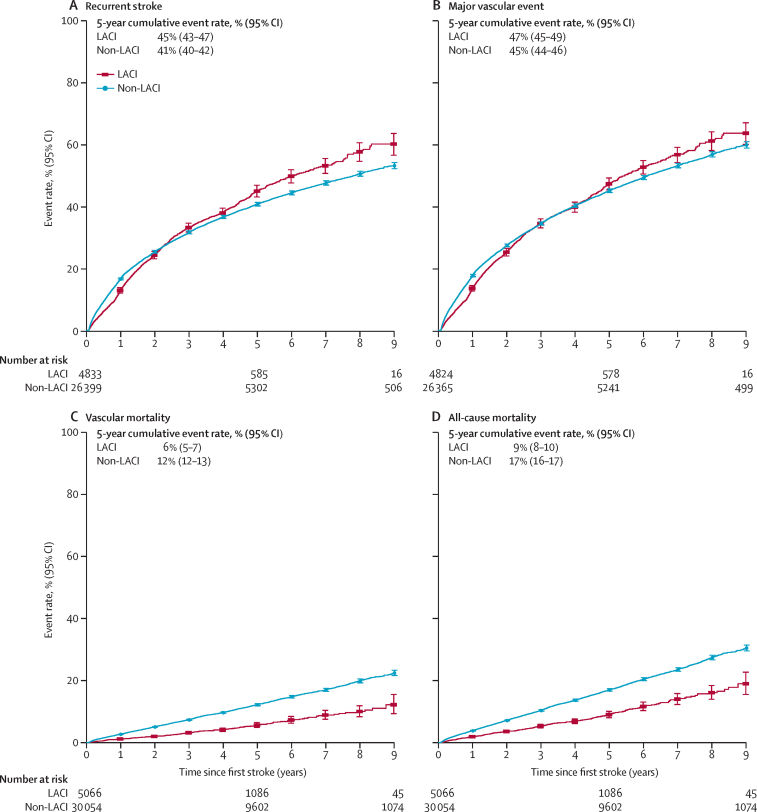
Estimated cumulative event rates of recurrent stroke, major vascular events, vascular mortality, and all-cause mortality from 28 days after first ischaemic stroke of different subtypes Plotted lines show the cumulative incidence, starting with the date of first stroke. Deaths from any cause were treated as competing risks. Participants experiencing an event or death within 28 days following a first stroke were excluded. Only participants whose first stroke was ischaemic stroke were included. LACI=lacunar infarction.

Among those who survived a first stroke, the cumulative recurrence rates from 28 days after the first stroke were 17% (95% CI 16–17) at 1 year, 41% (41–42) at 5 years, and 53% (52–54) at 9 years (table 2). All-cause mortality (predominantly from vascular causes) was 4% (4–4) at 1 year, 17% (17–18) at 5 years, and 30% (30–31) at 9 years. Individuals with intracerebral haemorrhage had higher post-28-day mortality than any other stroke type, with mortality rates of 11% (10–12) at 1 year, 28% (26–29) at 5 years, and 41% (39–44) at 9 years (appendix p 6), compared with 4% (3–4) at 1 year, 16% (15–16) at 5 years, and 29% (28–30) at 9 years for those with ischaemic stroke (appendix p 5). At 5 years, the all-cause mortality for those with intracerebral haemorrhage (28% [26–29]) was almost twice as high as that for other stroke types (figure 2). Stroke recurrence rates at 5 years were similar for those with intracerebral haemorrhage (44% [42–46]), ischaemic stroke (41% [41–42]), and unspecified stroke type (40% [35–44]), but were lower for those with subarachnoid haemorrhage (22% [18–27]). Mortality at 5 years was almost twice as high for individuals with non-lacunar infarction compared with lacunar infarction (17% [16–17] *vs* 9% [8–10]), and the stroke recurrence rates were similar (41% [40–42] *vs* 45% [43–47]; figure 3).

**Table 2:** Cumulative event rate of recurrent stroke, major vascular events, vascular mortality, and all-cause mortality (n=45 732)

	**28 days**	**1 year**	**2 years**	**3 years**	**4 years**	**5 years**	**6 years**	**7 years**	**8 years**	**9 years**
**Recurrent stroke**										
Events	0	5616	8140	9685	10 623	11 298	11 722	11 995	12 161	12 230
No events or death	35 654	24 916	18 535	13 723	9953	7008	4799	2937	1565	668
Deaths	0	655	1067	1391	1634	1786	1910	2002	2064	2077
Censored	0	4467	7912	10 855	13 444	15 562	17 223	18 720	19 864	20 679
Cumulative event rate, % (95% CI)	0	17% (16–17)	26% (25–26)	32% (31–33)	37% (36–37)	41% (41–42)	45% (44–45)	48% (47–49)	51% (50–52)	53% (52–54)
Major vascular event*										
Events	0	5963	8731	10 461	11 552	12 324	12 816	13 148	13 346	13 426
No events or death	35 605	24 839	18 448	13 646	9872	6933	4738	2894	1547	660
Deaths	0	347	548	698	807	877	937	975	1001	1005
Censored	0	4456	7878	10 800	13 374	15 471	17 114	18 588	19 711	20 514
Cumulative event rate, % (95% CI)	0	18% (17–18)	27% (27–28)	35% (34–35)	40% (40–41)	45% (45–46)	49% (49–50)	53% (53–54)	57% (56–58)	60% (59–61)
**Vascular mortality**										
Events	0	1208	1972	2597	3113	3545	3885	4105	4284	4362
No events or death	40 370	33 334	27 525	22 097	17 001	12 562	9078	5984	3321	1368
Deaths	0	426	729	962	1168	1298	1416	1496	1550	1569
Censored	0	5402	10 144	14 714	19 088	22 965	25 991	28 785	31 215	33 071
Cumulative event rate, % (95% CI)	0	3% (3–3)	6% (5–6)	8% (8–8)	10% (10–10)	13% (12–13)	15% (15–16)	17% (17–18)	20% (20–21)	23% (22–24)
**All-cause mortality**										
Events	0	1634	2701	3559	4281	4843	5301	5601	5834	5931
No events or death	40 370	33 334	27 525	22 097	17 001	12 562	9078	5984	3321	1368
Censored	0	5402	10 144	14 714	19 088	22 965	25 991	28 785	31 215	33 071
Cumulative event rate, % (95% CI)	0	4% (4–4)	8% (7–8)	11% (10–11)	14% (14–14)	17% (17–18)	21% (20–21)	24% (23–24)	27% (27–28)	30% (30–31)

Time-points are time since first stroke.

* Stroke, myocardial infarction, and vascular mortality.

For all types of first stroke, the cumulative recurrent stroke rate at 5 years was higher in men than women (except for subarachnoid haemorrhage) and in urban than rural areas (appendix p 14). All-cause mortality at 5 years was higher in men than women (except for subarachnoid haemorrhage), but lower in urban than rural areas for all stroke types (appendix p 15).

After a first ischaemic stroke, 91% of recurrent strokes were ischaemic stroke (urban 94%, rural 86%), 7% were intracerebral haemorrhage (urban 3%, rural 11%), and 2% were unspecified stroke type (urban 2%, rural 2%; appendix p 10). After a first intracerebral haemorrhage, 56% of recurrent strokes were intracerebral haemorrhage (urban 43%, rural 62%), 41% were ischaemic stroke (urban 53%, rural 35%), and 3% were unspecified stroke type (urban 3%, rural 3%). After a first subarachnoid haemorrhage, 43% of recurrent strokes were ischaemic stroke, 29% were intracerebral haemorrhage, and 27% were subarachnoid haemorrhage. After a stroke of unspecified type, 69% of recurrent strokes were ischaemic stroke, 22% were unspecified stroke, and 9% were intracerebral haemorrhage (appendix p 10).

After a first non-lacunar infarction, 80% of recurrent strokes were non-lacunar infarction (urban 78%, rural 82%), 10% were lacunar infarction, and 7% were intracerebral haemorrhage (appendix p 10). After a lacunar infarction, 47% of recurrent strokes were lacunar infarction (urban 50%, rural 23%), 48% were non-lacunar infarction (urban 47%, rural 64%), and 3% were intracerebral haemorrhage (urban 2%, rural 13%).

Compared with recurrent stroke (41%), the event rate of myocardial infarction at 5 years after first stroke was much lower, at about 2% (appendix p 16). The 5-year event rate for non-myocardial-infarction cardiac events was 15% after ischaemic stroke and 7% after intracerebral haemorrhage.

Among individuals who survived a first stroke at 28 days, the cumulative event rates for major vascular events (ie, non-fatal recurrent stroke, non-fatal myocardial infarction, or vascular death) were 18% at 1 year, 45% at 5 years, and 60% at 9 years (table 2). After combining both short-term (≤28 days) and long-term (>28 days) risks, the cumulative mortality after a first stroke was 15% at 1 year (ischaemic stroke 7%, intracerebral haemorrhage 53%) and 26% at 5 years (ischaemic stroke 19%, intracerebral haemorrhage 62%; appendix pp 8, 9).

After excluding individuals with stroke as secondary diagnosis, analyses restricted to 21 817 individuals with an adjudicated diagnosis of a non-fatal first stroke event as their main diagnosis showed similar 5-year cumulative event rates for recurrent stroke, major vascular events, vascular and all-cause mortality (appendix p 11). Further sensitivity analyses excluding individuals with recurrent stroke events who also had comorbidities only slightly reduced the overall 5-year recurrence rates (from 41% to 35%; appendix p 12).

## Discussion

In this large community-based study in China, the incidence of stroke in the adult population was high, with around 9% of included participants experiencing a stroke during 9-year follow-up. Prognosis was also notably poor, with one in ten individuals dying within 28 days after onset of a first stroke, almost one in five dying by 5 years and more than 40% experiencing a recurrent stroke within 5 years of a first stroke. Among stroke types, the short-term and long-term prognosis for individuals with intracerebral haemorrhage was substantially worse than for those with ischaemic stroke, particularly in rural areas. After a first ischaemic stroke, although the 28-day mortality was low, the long-term risks of recurrent stroke and mortality were high. After a first ischaemic stroke, most of the incidences of recurrent stroke were also ischaemic stroke, whereas after a first intracerebral haemorrhage, a higher proportion of recurrent strokes were ischaemic stroke than were intracerebral haemorrhage.

The event rate of first stroke in the present study population was considerably higher than the incidence reported in a recent national representative survey (345·1 per 100 000 person-years).^3^ The discrepant results in the present study might reflect the older mean age of the CKB study participants or the inclusion of areas with a high incidence of stroke (eg, Harbin and Hunan).^11^ Because the present study was not designed to be nationally representative, it is not appropriate to make direct comparisons of stroke incidence in CKB with those in representative national surveys. Nevertheless, the observed north-to-south gradients and rural-urban differences in stroke incidence observed in CKB were generally consistent with those reported in the most recent representative national survey.^3^

In the present study, 28-day mortality of intracerebral haemorrhage was considerably higher than that of ischaemic stroke (47% *vs* 3%), which is expected and could reflect differences in both pathology and effects of treatment.^17^ A systematic review of 8145 individuals with intracerebral haemorrhage from 36 studies in 21 countries also reported a similar 28-day mortality of 40%,^18^ and a previous study involving 16 031 Chinese adults who were enrolled between 1991 and 2000 reported 28-day mortality of 49·4% for intracerebral haemorrhage.^19^ Similar studies in western populations have reported lower 28-day mortality of intracerebral haemorrhage (eg, 29·6% in France^20^ and 30·7% in Sweden^21^). The higher mortality of intracerebral haemorrhage in China might reflect differences in stroke severity (due to poor detection and control of hypertension in China),^11^, ^22^ or delays in access to hospital care (including access to neurosurgery), particularly in rural areas.^11^ Similarly, differences in lifestyle factors might also account for the higher mortality observed in rural areas than urban areas in CKB. Unexpectedly, the present study showed lower mortality for subarachnoid haemorrhage than for intracerebral haemorrhage. Because subarachnoid haemorrhage is relatively rare among stroke types, the possibility of misclassification of subarachnoid haemorrhage as intracerebral haemorrhage for some individuals cannot be entirely excluded. The lower mortality of subarachnoid haemorrhage in Asian countries (Japan and China) than in other regions was also reported in a recent meta-analysis of subarachnoid haemorrhage involving 33 studies in 19 countries recruited after 1995.^23^ In contrast with intracerebral haemorrhage, the 28-day mortality for ischaemic stroke observed in the present study and in previous studies in China were considerably lower than those reported in western populations,^21^, ^24^, ^25^, ^26^ possibly reflecting differences in the incidence of ischaemic stroke subtypes (eg, a higher proportion of individuals with small vessel ischaemic stroke) or better detection of less severe stroke types.^27^, ^28^

Few previous studies in China have examined the long-term prognosis after the onset of first stroke. In the China National Stroke Registry study, which involved 11 560 individuals with ischaemic stroke recruited in 2007–08 from major hospitals, 1-year cumulative mortality after a first ischaemic stroke was twice as high as that reported in the present study (14% *vs* 7%),^29^ but the stroke recurrence rate at 1 year was comparable to that observed in the present study (17% *vs* 16%). Less evidence is available on the long-term prognosis after first intracerebral haemorrhage. In a meta-analysis of 2408 individuals with intracerebral haemorrhage from 122 studies in different countries,^30^ the overall cumulative mortality was 54% at 1 year and 71% at 5 years. The results of the present study were similar to those observed in a recent Swedish study, which reported mortality rates of 42% at 1 year and 62% at 5 years.^21^ A meta-analysis involving 1102 individuals with intracerebral haemorrhage enrolled between 1981 and 2007 reported higher stroke recurrence rates than the present study (46% *vs* 23%), but the differences might reflect improvements in health-care during the period between the studies.^30^

The poor long-term prognosis after a first stroke in China might also reflect the inadequate use of established medications (eg, antiplatelet therapy, blood pressure-lowering, and lipid-lowering treatments), as shown in a previous report from the CKB study population.^22^ Other community-based studies in China have reported that only 10% of individuals with ischaemic stroke used aspirin, 2% used LDL cholesterol-lowering therapy, and around 35% used antihypertensive medication.^11^, ^31^ The scarce use of such treatments with proven efficacy might reflect inadequate reimbursement policies, poor access to primary health care services,^32^ or poor awareness among patients and their doctors of the need for long-term use of such treatments for stroke survivors.^11^, ^33^

The present study showed that nearly all incidences of recurrent stroke after ischaemic stroke were also ischaemic stroke, which was consistent with previous studies done in western populations.^34^ In contrast with previous reports that suggested that lacunar infarction and non-lacunar infarction have a distinct pathology,^28^, ^35^ the present study showed that subsequent stroke can be both lacunar infarction and non-lacunar infarction after first lacunar infarction or non-lacunar infarction indicating overlapping causes underlying lacunar infarction and non-lacunar infarction. Few large studies have specifically examined the type of recurrent stroke events after intracerebral haemorrhage. A systematic review of ten studies, involving 1880 individuals with intracerebral haemorrhage recorded between 1982 and 2000, reported that 75% of the individuals who had recurrent strokes had intracerebral haemorrhage.^36^ By contrast, a meta-analysis involving 2408 individuals with intracerebral haemorrhage reported similar proportions of recurrent ischaemic stroke and recurrent intracerebral haemorrhage after first intracerebral haemorrhage.^30^ The present study, involving three-times as many individuals with intracerebral haemorrhage as the previous meta-analysis,^30^ also reported that more than 40% of recurrent strokes after intracerebral haemorrhage were ischaemic stroke.

Current guidelines recommend use of antiplatelet therapy and statins for ischaemic stroke, but not for intracerebral haemorrhage.^37^ However, most of the available evidence about the use of such treatment in intracerebral haemorrhage is based on studies done in western populations in which the incidence of intracerebral haemorrhage was low. Given the high incidence of intracerebral haemorrhage in China, large randomised trials are needed to assess the efficacy and safety of long-term antiplatelet and cholesterol-lowering therapy for secondary prevention of ischaemic stroke. A previous estimate suggested that the benefits of LDL cholesterol-lowering treatments on ischaemic stroke and ischaemic heart disease would exceed the adverse effects on intracerebral haemorrhage by more than five times in Chinese adults.^38^

This study had several strengths, including the prospective study design, the large number of participants, detailed data on stroke types, and complete long-term follow-up for both fatal and non-fatal incidences of stroke and other vascular diseases. Of note, more than 90% of the reported first strokes were confirmed by brain imaging. However, the study also had several limitations. Firstly, the CKB cohort was established mainly to investigate the determinants of common chronic diseases. Therefore, the study design focused on diversity rather than representativeness of study populations. As such, the findings may not be directly generalisable to the overall Chinese population. Secondly, recurrent strokes were defined as any reported stroke occurring more than 24 h after a first incident stroke that led to new hospitalisation.^35^ It is possible that some of the recurrent stroke cases might not be new stroke events, but were recorded as new cases for various reasons (including transfers between different hospitals). However, most of the recurrent strokes occurred many months or years after their initial event, frequently involving a different stroke pathological type, suggesting that they were unlikely to be readmissions for the same stroke or an evolving stroke after a first event. Thirdly, stroke survivors admitted to hospitals subsequently for reasons other than a new stroke could still have a stroke diagnosis recorded in their medical records, leading to false positive diagnoses for recurrent strokes. However, sensitivity analyses using recurrent stroke as the primary diagnosis or excluding those that also had comorbidities recorded at hospital admission did not notably alter the high rates of stroke recurrence and mortality. Nevertheless, the possibility of overestimation or underestimation of stroke recurrence after a first stroke cannot be entirely excluded and the verification of at least some recurrent stroke events in future studies would help to clarify this.

This study showed a high incidence of stroke in Chinese adults, and a surprisingly poor long-term prognosis after a first stroke of any type, particularly for individuals living in rural areas. The findings highlight the need for improved primary prevention of stroke through lifestyle modification, and for urgent improvements to the delivery of effective secondary prevention treatments for stroke (eg, anti-platelet, lipid-lowering, and blood pressure-lowering medication) in both urban and rural areas in China and in other low-income and middle-income countries.
