# Supplementary material for: Mortality and recurrent vascular events after first incident stroke: a 9-year community-based study of 0·5 million Chinese adults
Source: Lancet Glob Health. 2020 Mar 18;8(4):e580–90. doi: 10.1016/S2214-109X(20)30069-3 (PMC7090905; doi:10.1016/S2214-109X(20)30069-3)

THE LANCET
Global Health

Supplementary appendix

This appendix formed part of the original submission and has been peer reviewed. We post it as supplied by the authors.

Supplement to: Chen Y, Wright N, Guo Y, et al. Mortality and recurrent vascular events after first incident stroke: a 9-year community-based study of 0·5 million Chinese adults. *Lancet Glob Health* 2020; **8:** e580–90.

Supplementary Appendix

Supplement to: Yiping Chen DPhil, Neil Wright PhD, Yu Guo MSc, Iain Turnbull MRCP, Christiana Kartsonaki DPhil, Ling Yang PhD, Zheng Bian MSc, Pei Pei MSc, Dongxia Pan MBBS, Yidan Zhang BSc, Haiqiang Qin MD, Yilong Wang MD, Jun Lv PhD, Ming Liu MD, Zilong Hao,Yongjun Wang MD, Canqing Yu PhD, Richard Peto FRS, Rory Collins FRS, Liming Li MPH, Robert Clarke FRCP, Zhengming Chen DPhil, for the China Kadoorie Biobank Collaborative Group. Mortality and recurrent vascular events after first incident stroke: a 9-year community-based study of 0.5 million Chinese adults

Table of Contents:

2	Table of contents	
3	Members of the China Kadoorie Biobank Collaborative Group	
4	webtable 1:	Standardised event rates (95% CIs) per 100,000 person-years of different types of stroke after excluding 23,129 participants with prior IHD and/or stroke/TIA
5	webtable 2:	Cumulative event rate of recurrent stroke, major vascular event, vascular mortality and all-cause mortality from 28 days after first IS
6	webtable 3:	Cumulative event rate of recurrent stroke, major vascular event, vascular mortality and all-cause mortality from 28 days after first ICH
7	webtable 4:	Cumulative event rate of recurrent stroke, major vascular event, vascular mortality and all-cause mortality following first stroke (combining before and after 28 days)
8	webtable 5:	Cumulative event rate of recurrent stroke, major vascular event, vascular mortality and all-cause mortality following first IS (combining before and after 28 days)
9	webtable 6:	Cumulative event rate of recurrent stroke, major vascular event, vascular mortality and all-cause mortality following first ICH (combining before and after 28 days)
10	webtable 7:	Change of pathological type in the recurrent stroke up to 5 years following first stroke
10	webtable 8:	Change of pathological type in the recurrent stroke up to 5 years following first ischemic stroke subtypes
11	webtable 9:	Cumulative event rate of recurrent stroke, major vascular events, vascular mortality and all-cause mortality from 28 days following first stroke among 21,817 adjudicated non-fatal events
12	webtable 10:	Cumulative event rate of recurrent stroke following first stroke excluding recurrent stroke events with co-morbidity
13	webfigure 1:	Age−specific 28−day mortality rate following first stroke of different types by (a) sex and (b) area
14	webfigure 2:	Estimated cumulative event rate of recurrent stroke from 28 days after first stroke of different types by (a) sex and (b) area
15	webfigure 3:	Estimated cumulative all−cause mortality rate from 28 days after first stroke of different types by (a) sex and (b) area
16	webfigure 4:	Estimated cumulative event rate of MI and other cardiac diseases from 28 days after first stroke of different types

Members of the China Kadoorie Biobank Collaborative Group

International Steering Committee: Junshi Chen, Zhengming Chen (PI), Robert Clarke, Rory Collins, Yu Guo, Liming Li (PI), Jun Lv, Richard Peto, and Robin Walters.
International Co-ordinating Centre, Oxford: Daniel Avery, Derrick Bennett, Ruth Boxall, Fiona Bragg, Yumei Chang, Yiping Chen, Zhengming Chen, Robert Clarke, Huaidong Du, Simon Gilbert, Alex Hacker, Michael Holmes, Christiana Kartsonaki, Rene Kerosi, Garry Lancaster, Kuang Lin, John McDonnell, Iona Millwood, Qunhua Nie, Paul Ryder, Sam Sansome, Dan Schmidt, Rajani Sohoni, Iain Turnbull, Robin Walters, Jenny Wang, Lin Wang, Neil Wright, Ling Yang, and Xiaoming Yang.

National Co-ordinating Centre, Beijing: Zheng Bian, Yu Guo, Xiao Han, Can Hou, Biao Jing, Chao Liu, Jun Lv, Pei Pei, and Canqing Yu.

Regional Co-ordinating Centres: Qingdao **Qingdao** CDC: Zengchang Pang, Ruqin Gao, Shanpeng Li, Shaojie Wang, Yongmei Liu, Ranran Du, Yajing Zang, Liang Cheng, Xiaocao Tian, Hua Zhang, Yaoming Zhai, Feng Ning, Xiaohui Sun, Feifei Li. Licang CDC: Silu Lv, Junzheng Wang, Wei Hou. **Heilongjiang** Provincial CDC: Mingyuan Zeng, Ge Jiang, Xue Zhou. Nangang CDC: Liqiu Yang, Hui He, Bo Yu, Yanjie Li, Qinai Xu, Quan Kang, Ziyan Guo. **Hainan** Provincial CDC: Dan Wang, Ximin Hu, Hongmei Wang, Jinyan Chen, Yan Fu, Zhenwang Fu, Xiaohuan Wang. Meilan CDC: Min Weng, Zhendong Guo, Shukuan Wu, Yilei Li, Huimei Li, Zhifang Fu. **Jiangsu** Provincial CDC: Ming Wu, Yonglin Zhou, Jinyi Zhou, Ran Tao, Jie Yang, Jian Su. Suzhou CDC: Fang Liu, Jun Zhang, Yihe Hu, Yan Lu, Liangcai Ma, Aiyu Tang, Shuo Zhang, Jianrong Jin, Jingchao Liu. **Guangxi** Provincial CDC: Zhenzhu Tang, Naying Chen, Ying Huang. Liuzhou CDC: Mingqiang Li, Jinhuai Meng, Rong Pan, Qilian Jiang, Jian Lan, Yun Liu, Liuping Wei, Liyuan Zhou, Ningyu Chen, Ping Wang, Fanwen Meng, Yulu Qin, Sisi Wang. **Sichuan** Provincial CDC: Xianping Wu, Ningmei Zhang, Xiaofang Chen, Weiwei Zhou. Pengzhou CDC: Guojin Luo, Jianguo Li, Xiaofang Chen, Xunfu Zhong, Jiaqiu Liu, Qiang Sun. **Gansu** Provincial CDC: Pengfei Ge, Xiaolan Ren, Caixia Dong. Maiji CDC: Hui Zhang, Enke Mao, Xiaoping Wang, Tao Wang, Xi Zhang. **Henan** Provincial CDC: Ding Zhang, Gang Zhou, Shixian Feng, Liang Chang, Lei Fan. Huixian CDC: Yulian Gao, Tianyou He, Huarong Sun, Pan He, Chen Hu, Xukui Zhang, Huifang Wu, Pan He. **Zhejiang** Provincial CDC: Min Yu, Ruying Hu, Hao Wang. Tongxiang CDC: Yijian Qian, Chunmei Wang, Kaixu Xie, Lingli Chen, Yidan Zhang, Dongxia Pan, Qijun Gu. **Hunan** Provincial CDC: Yuelong Huang, Biyun Chen, Li Yin, Huilin Liu, Zhongxi Fu, Qiaohua Xu. Liuyang CDC: Xin Xu, Hao Zhang, Huajun Long, Xianzhi Li, Libo Zhang, and Zhe Qiu.

Web Table 1: Standardised* event rates (95% CIs) per 100,000 person-years of different types of stroke after excluding 23,129 participants with prior IHD and/or stroke/TIA

	Stroke type					Any stroke
	IS		ICH	SAH	Unspecified	
	LACI (n=5,130)	Non-LACI (n=31,458)	(n=7,440)	(n=702)	(n=1,002)	(n=45,732)
**Age at risk (years)**						
40 – 49	24 (21-27)	145 (139-151)	50 (46-53)			232 (223-240)
50 – 59	91 (86-96)	462 (452-473)	107 (102-112)	14 (12-16)	15 (14-17)	690 (677-703)
60 – 69	159 (152-167)	985 (966-1004)	209 (200-217)	21 (19-24)	26 (23-29)	1401 (1378-1423)
70 – 79	272 (258-286)	1959 (1921-1998)	482 (463-502)		63 (56-70)	2802 (2755-2848)
**Sex**						
Men	103 (99-108)	729 (717-742)	193 (187-199)			1062 (1047-1077)
Women	114 (110-118)	624 (615-634)	129 (125-133)			903 (891-914)
**Area**						
Rural	42 (39-45)	630 (620-639)	211 (205-217)	17 (15-18)	20 (18-21)	919 (907-931)
Urban	197 (190-203)	713 (701-725)	87 (82-91)			1031 (1017-1045)
**Highest education**						
No formal / primary school						
Middle school or higher		756 (739-774)	128 (121-136)			1068 (1047-1089)
**Annual household income (CNY)**						
<20,000	116 (112-121)	722 (712-733)	178 (173-183)			1055 (1042-1067)
20,000 – 34,9999		607 (592-623)				865 (847-884)
≥35,000		559 (541-577)				788 (767-809)
**Current smoker**						
Men Current smoker		773 (766-780)	211 (207-214)			1120 (1112-1129)
Not current smoker		740 (732-748)	197 (193-202)			1085 (1076-1095)
Women Current smoker						
Not current smoker	110 (108-113)	605 (599-611)	124 (121-126)			873 (866-880)
**Current alcohol drinker**						
Men Current drinker		759 (753-765)	188 (185-191)			1096 (1089-1104)
Not current drinker		764 (754-775)				1119 (1106-1131)
Women Current drinker		681 (671-692)	110 (105-114)			1001 (988-1014)
Not current drinker	83 (80-85)	568 (562-575)	130 (127-133)			814 (806-822)
**Prevalent disease**						
Prevalent diabetes						
No prevalent diabetes	108 (105-111)	643 (636-651)	154 (150-158)			941 (931-950)
Prevalent hypertension		1273 (1237-1309)				1850 (1805-1894)
No prevalent hypertension	109 (106-113)	600 (592-608)	134 (131-138)			876 (867-885)
**All**	110 (107-113)	668 (661-676)	156 (153-160)	15 (14-16)	21 (20-22)	970 (961-979)

4.46 million person-years follow up

* Event rates standardised by age, sex and rural/urban as appropriate to the 40 to 79 year old CKB population excluding participants with prior IHD and/or stroke/TIA.

Where any strata contain less than 20 events, standardised rates are not presented.

Web Table 2: Cumulative event rate of recurrent stroke, major vascular event, vascular mortality and all-cause mortality from 28 days after first IS

	Years since IS (n=36,588)									
	**28 days**	**1**	**2**	**3**	**4**	**5**	**6**	**7**	**8**	**9**
Recurrent stroke										
No. events	0	4,708	6,965	8,345	9,185	9,766	10,137	10,368	10,505	10,569
No. free of any events and deaths	31,232	21,877	16,155	11,897	8,515	5,887	3,994	2,390	1,260	522
No. deaths	0	541	892	1,167	1,379	1,512	1,618	1,694	1,746	1,758
No. censored	0	4,106	7,220	9,823	12,153	14,067	15,483	16,780	17,721	18,383
Cumulative event rate, % (95% CI)	0	16 (16 - 17)	25 (25 - 26)	32 (31 - 32)	37 (36 - 37)	41 (41 - 42)	45 (44 - 46)	48 (47 - 49)	51 (50 - 52)	54 (53 - 55)
Major vascular event *										
No. events	0	4,995	7,470	9,005	9,982	10,647	11,077	11,355	11,517	11,591
No. free of any events and deaths	31,189	21,807	16,073	11,825	8,441	5,819	3,939	2,351	1,244	515
No. deaths	0	290	456	586	678	741	791	825	848	852
No. censored	0	4,097	7,190	9,773	12,088	13,982	15,382	16,658	17,580	18,231
Cumulative event rate, % (95% CI)	0	17 (17 - 18)	27 (27 - 28)	34 (34 - 35)	40 (40 - 41)	45 (45 - 46)	50 (49 - 50)	54 (53 - 54)	57 (56 - 58)	60 (59 - 61)
Vascular mortality										
No. events	0	812	1,416	1,921	2,342	2,699	2,978	3,152	3,292	3,359
No. free of any events and deaths	35,120	29,037	23,936	19,173	14,653	10,688	7,683	5,001	2,761	1,119
No. deaths	0	357	617	822	1,001	1,114	1,212	1,282	1,331	1,345
No. censored	0	4,914	9,151	13,204	17,124	20,619	23,247	25,685	27,736	29,297
Cumulative event rate, % (95% CI)	0	2 (2 - 3)	5 (4 - 5)	7 (6 - 7)	9 (9 - 9)	11 (11 - 12)	14 (13 - 14)	16 (15 - 17)	19 (18 - 20)	21 (20 - 22)
All-cause mortality										
No. events	0	1,169	2,033	2,743	3,343	3,813	4,190	4,434	4,623	4,704
No. free of any events and deaths	35,120	29,037	23,936	19,173	14,653	10,688	7,683	5,001	2,761	1,119
No. censored	0	4,914	9,151	13,204	17,124	20,619	23,247	25,685	27,736	29,297
Cumulative event rate, % (95% CI)	0	4 (3 - 4)	7 (6 - 7)	10 (9 - 10)	13 (12 - 13)	16 (15 - 16)	19 (19 - 20)	22 (22 - 23)	26 (25 - 27)	29 (28 - 30)

* Stroke, MI and vascular mortality

Web Table 3: Cumulative event rate of recurrent stroke, major vascular event, vascular mortality and all-cause mortality from 28 days after first ICH

	Years since ICH (n=7,440)									
	**28 days**	**1**	**2**	**3**	**4**	**5**	**6**	**7**	**8**	**9**
Recurrent stroke										
No. events	0	741	957	1,091	1,166	1,231	1,275	1,308	1,332	1,335
No. free of any events and deaths	3,262	2,180	1,693	1,301	1,024	806	579	378	196	86
No. deaths	0	89	135	174	202	217	230	243	249	250
No. censored	0	252	477	696	870	1,008	1,178	1,333	1,485	1,591
Cumulative event rate, % (95% CI)	0	23 (22 - 25)	31 (29 - 33)	37 (35 - 38)	40 (38 - 42)	44 (42 - 46)	47 (45 - 49)	50 (47 - 52)	53 (50 - 55)	53 (51 - 56)
Major vascular event *										
No. events	0	787	1,024	1,180	1,269	1,345	1,395	1,438	1,466	1,470
No. free of any events and deaths	3,258	2,175	1,689	1,296	1,018	799	573	374	194	85
No. deaths	0	44	69	87	102	107	115	118	120	120
No. censored	0	252	476	695	869	1,007	1,175	1,328	1,478	1,583
Cumulative event rate, % (95% CI)	0	25 (23 - 26)	33 (32 - 35)	40 (38 - 41)	44 (42 - 46)	48 (46 - 50)	51 (49 - 54)	55 (53 - 57)	59 (56 - 61)	60 (57 - 63)
Vascular mortality										
No. events	0	348	487	590	672	732	782	820	852	859
No. free of any events and deaths	3,930	3,173	2,643	2,161	1,726	1,387	1,036	712	383	159
No. deaths	0	54	87	113	132	144	158	166	170	173
No. censored	0	355	713	1,066	1,400	1,667	1,954	2,232	2,525	2,739
Cumulative event rate, % (95% CI)	0	9 (8 - 10)	13 (12 - 14)	17 (16 - 18)	20 (19 - 21)	23 (21 - 24)	26 (24 - 27)	29 (27 - 31)	32 (30 - 35)	34 (32 - 36)
All-cause mortality										
No. events	0	402	574	703	804	876	940	986	1,022	1,032
No. free of any events and deaths	3,930	3,173	2,643	2,161	1,726	1,387	1,036	712	383	159
No. censored	0	355	713	1,066	1,400	1,667	1,954	2,232	2,525	2,739
Cumulative event rate, % (95% CI)	0	11 (10 - 12)	16 (14 - 17)	20 (19 - 21)	24 (23 - 26)	28 (26 - 29)	31 (29 - 33)	35 (33 - 37)	39 (37 - 41)	41 (39 - 44)

* Stroke, MI and vascular mortality

Web Table 4: Cumulative event rate of recurrent stroke, major vascular event, vascular mortality and all-cause mortality following first stroke (combining before and after 28 days)

	Years since first stroke (n=45,732)									
	**0**	**1**	**2**	**3**	**4**	**5**	**6**	**7**	**8**	**9**
Recurrent stroke										
No. events	0	11,457	13,981	15,526	16,464	17,139	17,563	17,836	18,002	18,071
No. free of any events and deaths	45,732	24,916	18,535	13,723	9,953	7,008	4,799	2,937	1,565	668
No. deaths	0	4,627	5,039	5,363	5,606	5,758	5,882	5,974	6,036	6,049
No. censored	0	4,732	8,177	11,120	13,709	15,827	17,488	18,985	20,129	20,944
Cumulative event rate, % (95% CI)	0	26 (26 - 26)	33 (32 - 33)	38 (37 - 38)	42 (41 - 42)	45 (45 - 46)	48 (47 - 48)	50 (50 - 51)	53 (52 - 53)	55 (54 - 55)
Major vascular event *										
No. events	0	15,825	18,593	20,323	21,414	22,186	22,678	23,010	23,208	23,288
No. free of any events and deaths	45,732	24,839	18,448	13,646	9,872	6,933	4,738	2,894	1,547	660
No. deaths	0	347	548	698	807	877	937	975	1,001	1,005
No. censored	0	4,721	8,143	11,065	13,639	15,736	17,379	18,853	19,976	20,779
Cumulative event rate, % (95% CI)	0	36 (35 - 36)	43 (43 - 44)	49 (48 - 49)	53 (53 - 54)	57 (57 - 58)	60 (60 - 61)	63 (63 - 64)	66 (66 - 67)	68 (68 - 69)
Vascular mortality										
No. events	0	6,291	7,055	7,680	8,196	8,628	8,968	9,188	9,367	9,445
No. free of any events and deaths	45,732	33,334	27,525	22,097	17,001	12,562	9,078	5,984	3,321	1,368
No. deaths	0	426	729	962	1,168	1,298	1,416	1,496	1,550	1,569
No. censored	0	5,681	10,423	14,993	19,367	23,244	26,270	29,064	31,494	33,350
Cumulative event rate, % (95% CI)	0	14 (14 - 14)	16 (16 - 16)	18 (18 - 18)	20 (20 - 20)	22 (22 - 23)	25 (24 - 25)	27 (26 - 27)	29 (28 - 30)	31 (31 - 32)
All-cause mortality										
No. events	0	6,717	7,784	8,642	9,364	9,926	10,384	10,684	10,917	11,014
No. free of any events and deaths	45,732	33,334	27,525	22,097	17,001	12,562	9,078	5,984	3,321	1,368
No. censored	0	5,681	10,423	14,993	19,367	23,244	26,270	29,064	31,494	33,350
Cumulative event rate, % (95% CI)	0	15 (15 - 15)	18 (17 - 18)	21 (20 - 21)	24 (23 - 24)	26 (26 - 27)	29 (29 - 30)	32 (32 - 33)	36 (35 - 36)	38 (37 - 39)

* Stroke, MI and vascular mortality

Web Table 5: Cumulative event rate of recurrent stroke, major vascular event, vascular mortality and all-cause mortality following first IS (combining before and after 28 days)

	Years since first IS (n=36,588)									
	**0**	**1**	**2**	**3**	**4**	**5**	**6**	**7**	**8**	**9**
Recurrent stroke										
No. events	0	8,908	11,165	12,545	13,385	13,966	14,337	14,568	14,705	14,769
No. free of any events and deaths	36,588	21,877	16,155	11,897	8,515	5,887	3,994	2,390	1,260	522
No. deaths	0	1,454	1,805	2,080	2,292	2,425	2,531	2,607	2,659	2,671
No. censored	0	4,349	7,463	10,066	12,396	14,310	15,726	17,023	17,964	18,626
Cumulative event rate, % (95% CI)	0	25 (25 - 26)	33 (33 - 34)	39 (38 - 39)	43 (43 - 44)	47 (46 - 48)	50 (50 - 51)	53 (52 - 54)	56 (55 - 56)	58 (57 - 59)
Major vascular event *										
No. events	0	10,151	12,626	14,161	15,138	15,803	16,233	16,511	16,673	16,747
No. free of any events and deaths	36,588	21,807	16,073	11,825	8,441	5,819	3,939	2,351	1,244	515
No. deaths	0	290	456	586	678	741	791	825	848	852
No. censored	0	4,340	7,433	10,016	12,331	14,225	15,625	16,901	17,823	18,474
Cumulative event rate, % (95% CI)	0	29 (28 - 29)	37 (37 - 38)	44 (43 - 44)	49 (48 - 49)	53 (52 - 54)	57 (56 - 57)	60 (59 - 61)	63 (63 - 64)	66 (65 - 67)
Vascular mortality										
No. events	0	2,025	2,629	3,134	3,555	3,912	4,191	4,365	4,505	4,572
No. free of any events and deaths	36,588	29,037	23,936	19,173	14,653	10,688	7,683	5,001	2,761	1,119
No. deaths	0	357	617	822	1,001	1,114	1,212	1,282	1,331	1,345
No. censored	0	5,169	9,406	13,459	17,379	20,874	23,502	25,940	27,991	29,552
Cumulative event rate, % (95% CI)	0	6 (5 - 6)	8 (8 - 8)	10 (10 - 10)	12 (12 - 12)	14 (14 - 15)	17 (16 - 17)	19 (18 - 19)	22 (21 - 22)	24 (23 - 25)
All-cause mortality										
No. events	0	2,382	3,246	3,956	4,556	5,026	5,403	5,647	5,836	5,917
No. free of any events and deaths	36,588	29,037	23,936	19,173	14,653	10,688	7,683	5,001	2,761	1,119
No. censored	0	5,169	9,406	13,459	17,379	20,874	23,502	25,940	27,991	29,552
Cumulative event rate, % (95% CI)	0	7 (6 - 7)	10 (9 - 10)	13 (12 - 13)	16 (15 - 16)	19 (18 - 19)	22 (21 - 23)	25 (24 - 26)	29 (28 - 29)	32 (31 - 33)

* Stroke, MI and vascular mortality

Web Table 6: Cumulative event rate of recurrent stroke, major vascular event, vascular mortality and all-cause mortality following first ICH (combining before and after 28 days)

	Years since first ICH (n=7,440)									
	**0**	**1**	**2**	**3**	**4**	**5**	**6**	**7**	**8**	**9**
Recurrent stroke										
No. events	0	2,132	2,348	2,482	2,557	2,622	2,666	2,699	2,723	2,726
No. free of any events and deaths	7,440	2,180	1,693	1,301	1,024	806	579	378	196	86
No. deaths	0	2,859	2,905	2,944	2,972	2,987	3,000	3,013	3,019	3,020
No. censored	0	269	494	713	887	1,025	1,195	1,350	1,502	1,608
Cumulative event rate, % (95% CI)	0	29 (28 - 30)	32 (31 - 33)	35 (34 - 36)	36 (35 - 38)	38 (37 - 39)	39 (38 - 40)	41 (39 - 42)	42 (41 - 43)	42 (41 - 44)
Major vascular event *										
No. events	0	4,952	5,189	5,345	5,434	5,510	5,560	5,603	5,631	5,635
No. free of any events and deaths	7,440	2,175	1,689	1,296	1,018	799	573	374	194	85
No. deaths	0	44	69	87	102	107	115	118	120	120
No. censored	0	269	493	712	886	1,024	1,192	1,345	1,495	1,600
Cumulative event rate, % (95% CI)	0	67 (66 - 68)	71 (70 - 72)	73 (72 - 74)	75 (74 - 76)	77 (76 - 78)	79 (78 - 80)	80 (79 - 81)	82 (81 - 83)	82 (81 - 84)
Vascular mortality										
No. events	0	3,840	3,979	4,082	4,164	4,224	4,274	4,312	4,344	4,351
No. free of any events and deaths	7,440	3,173	2,643	2,161	1,726	1,387	1,036	712	383	159
No. deaths	0	54	87	113	132	144	158	166	170	173
No. censored	0	373	731	1,084	1,418	1,685	1,972	2,250	2,543	2,757
Cumulative event rate, % (95% CI)	0	52 (51 - 53)	54 (53 - 55)	56 (55 - 57)	58 (56 - 59)	59 (58 - 60)	61 (59 - 62)	62 (61 - 63)	64 (63 - 65)	65 (63 - 66)
All-cause mortality										
No. events	0	3,894	4,066	4,195	4,296	4,368	4,432	4,478	4,514	4,524
No. free of any events and deaths	7,440	3,173	2,643	2,161	1,726	1,387	1,036	712	383	159
No. censored	0	373	731	1,084	1,418	1,685	1,972	2,250	2,543	2,757
Cumulative event rate, % (95% CI)	0	53 (51 - 54)	55 (54 - 56)	58 (56 - 59)	60 (59 - 61)	62 (60 - 63)	64 (62 - 65)	65 (64 - 67)	68 (66 - 69)	69 (67 - 70)

* Stroke, MI and vascular mortality

Web Table 7: Change of pathological type in the recurrent stroke up to 5 years following first stroke

	Second stroke type (%)			
	IS	ICH	SAH	Unspecified
**All**				
**First stroke type**				
IS	91	7	0	2
ICH	41	56	1	3
SAH	43	29	27	1
Unspecified	69	9	0	22
All	85	12	1	3
**Urban**				
**First stroke type**				
IS	94	3	0.3	2
ICH	53	43	1	3
SAH	54	21	25	0
Unspecified	73	3	0	24
All	91	6	0.5	3
**Rural**				
**First stroke type**				
IS	86	11	0.4	2
ICH	35	62	0.3	3
SAH	36	35	28	2
Unspecified	66	17	0	17
All	78	19	0.6	2

Percentages are calculated from estimated cumulative incidences, treating stroke types and all-cause mortality as competing risks.
E.g. In urban regions, up to five years after a first stroke that is IS, 94% of second strokes are also IS and 3% are ICH.

Web Table 8: Change of pathological type in the recurrent stroke up to 5 years following first ischemic stroke subtypes

	Second stroke type (%)				
	LACI	Non-LACI	ICH	SAH	Unspecified
**All**					
**First ischemic stroke type**					
LACI	47	48	3	0	1
Non-LACI	10	80	7	0	2
All	16	75	7	0	2
**Urban**					
**First ischemic stroke type**					
LACI	50	47	2	0.4	1
Non-LACI	16	78	4	0.3	3
All	24	71	3	0.3	2
**Rural**					
**First ischemic stroke type**					
LACI	23	64	13	0	0
Non-LACI	4	82	11	0.4	2
All	5	81	11	0.4	2

Web Table 9: Cumulative event rate of recurrent stroke, major vascular events, vascular mortality and all-cause mortality from 28 days following first stroke among 21,817 adjudicated non-fatal events

	Years since first stroke									
	**0**	**1**	**2**	**3**	**4**	**5**	**6**	**7**	**8**	**9**
Recurrent stroke										
No. events	0	2,954	4,716	5,922	6,675	7,184	7,514	7,716	7,829	7,868
No. free of any events and deaths	19,927	16,475	14,354	10,394	7,038	4,563	2,916	1,702	828	334
No. deaths	0	470	794	1,031	1,222	1,323	1,405	1,464	1,506	1,513
No. censored	0	28	63	2,580	4,992	6,857	8,092	9,045	9,764	10,212
Cumulative event rate, % (95% CI)	0	15 (14 - 15)	24 (23 - 24)	30 (30 - 31)	36 (35 - 36)	40 (40 - 41)	45 (44 - 46)	49 (48 - 50)	52 (51 - 53)	54 (53 - 56)
Major vascular event *										
No. events	0	3,200	5,128	6,464	7,325	7,900	8,275	8,512	8,643	8,688
No. free of any events and deaths	19,814	16,348	14,216	10,272	6,950	4,496	2,864	1,668	811	329
No. deaths	0	238	408	524	613	664	704	727	747	750
No. censored	0	28	62	2,554	4,926	6,754	7,971	8,907	9,613	10,047
Cumulative event rate, % (95% CI)	0	16 (16 - 17)	26 (25 - 27)	33 (33 - 34)	39 (39 - 40)	45 (44 - 46)	50 (49 - 51)	54 (53 - 55)	59 (57 - 60)	61 (60 - 62)
Vascular mortality										
No. events	0	688	1,218	1,666	2,032	2,282	2,455	2,588	2,693	2,737
No. free of any events and deaths	21,817	20,803	19,984	15,683	11,333	7,824	5,411	3,421	1,822	715
No. deaths	0	293	527	707	867	967	1,033	1,078	1,112	1,124
No. censored	0	33	88	3,761	7,585	10,744	12,918	14,730	16,190	17,241
Cumulative event rate, % (95% CI)	0	3 (3 - 3)	6 (5 - 6)	8 (7 - 8)	10 (10 - 11)	12 (12 - 13)	15 (14 - 15)	17 (16 - 18)	20 (19 - 21)	22 (21 - 24)
All-cause mortality										
No. events	0	981	1,745	2,373	2,899	3,249	3,488	3,666	3,805	3,861
No. free of any events and deaths	21,817	20,803	19,984	15,683	11,333	7,824	5,411	3,421	1,822	715
No. censored	0	33	88	3,761	7,585	10,744	12,918	14,730	16,190	17,241
Cumulative event rate, % (95% CI)	0	4 (4 - 5)	8 (8 - 8)	11 (11 - 12)	15 (14 - 15)	18 (17 - 18)	21 (20 - 21)	24 (23 - 25)	28 (27 - 29)	31 (30 - 32)

* Stroke, MI and vascular mortality

Web Table 10: Cumulative event rate of recurrent stroke following first stroke excluding recurrent stroke events with co-morbidity

	Years since first stroke (n=45,732)									
	**0**	**1**	**2**	**3**	**4**	**5**	**6**	**7**	**8**	**9**
Recurrent stroke										
No. events	0	4,694	6,851	8,209	9,051	9,660	10,037	10,264	10,413	10,481
No. free of any events and deaths	35,993	25,664	19,335	14,448	10,539	7,467	5,142	3,185	1,712	731
No. deaths	0	1,010	1,593	2,032	2,377	2,598	2,769	2,897	2,986	3,004
No. censored	0	4,625	8,214	11,304	14,026	16,268	18,045	19,647	20,882	21,777
Cumulative event rate, % (95% CI)	0	14 (13 - 14)	21 (21 - 22)	27 (26 - 27)	31 (31 - 32)	35 (35 - 36)	38 (38 - 39)	41 (40 - 42)	44 (43 - 44)	46 (45 - 47)

Second stroke events that occur on the same day as any other event (ICD-10 code not starting with I60, I61, I63, I64) are ignored. Cumulative event rate is from 28 days after first stroke.

Web Figure 1: Age−specific 28−day mortality rate following first stroke of different types by (a) sex and (b) area

(a) Sex

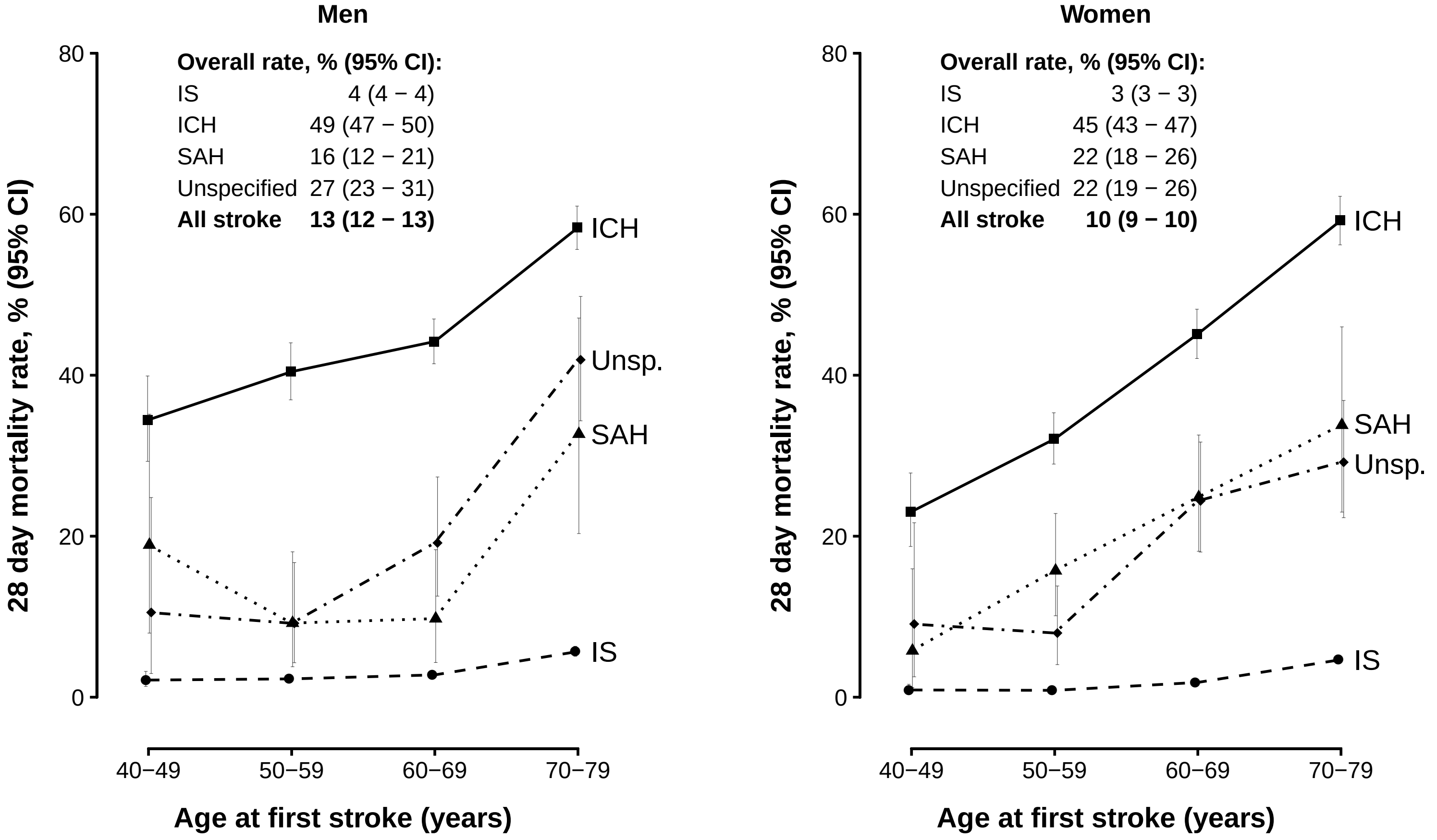

(b) Area

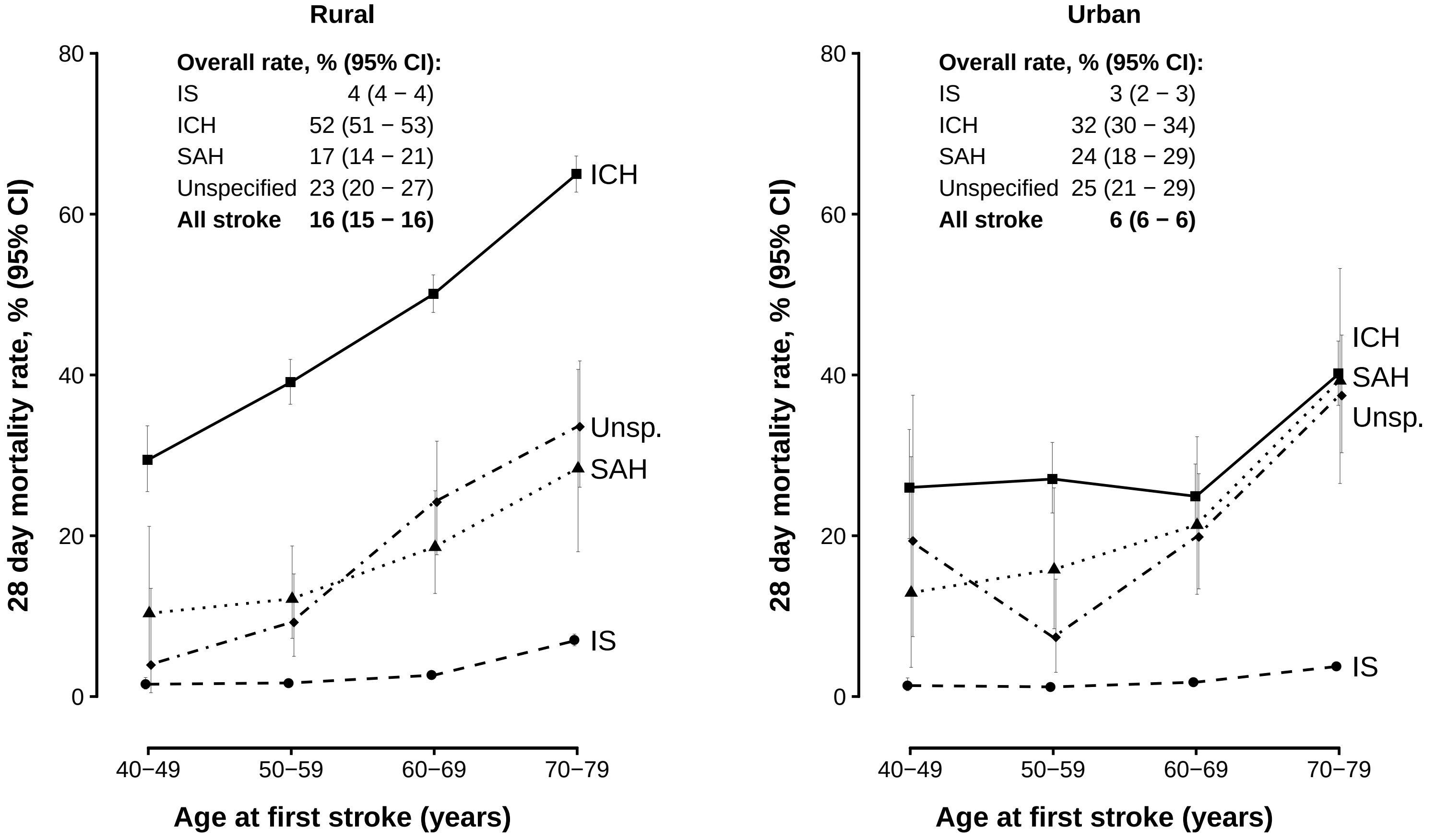

Web Figure 2: Estimated cumulative event rate of recurrent stroke from 28 days after first stroke of different types by (a) sex and (b) area

(a) Sex

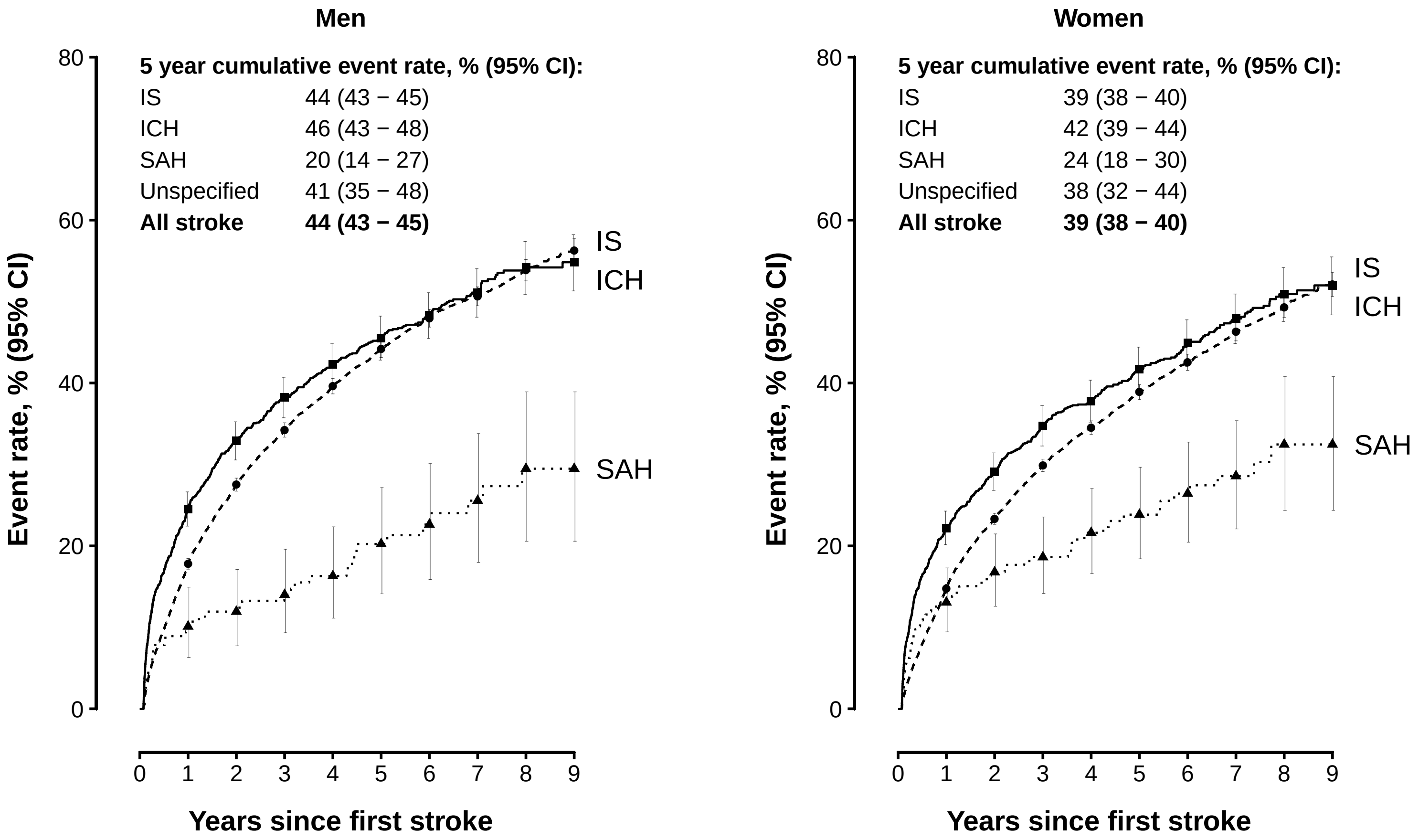

Men: Numbers at risk	0	5	9	
	13,770	2,538	211	IS
	1,657	391	38	ICH
	194	68	17	SAH

Women: Numbers at risk	0	5	9	
	17,462	3,349	311	IS
	1,605	415	48	ICH
	289	90	10	SAH

(b) Area

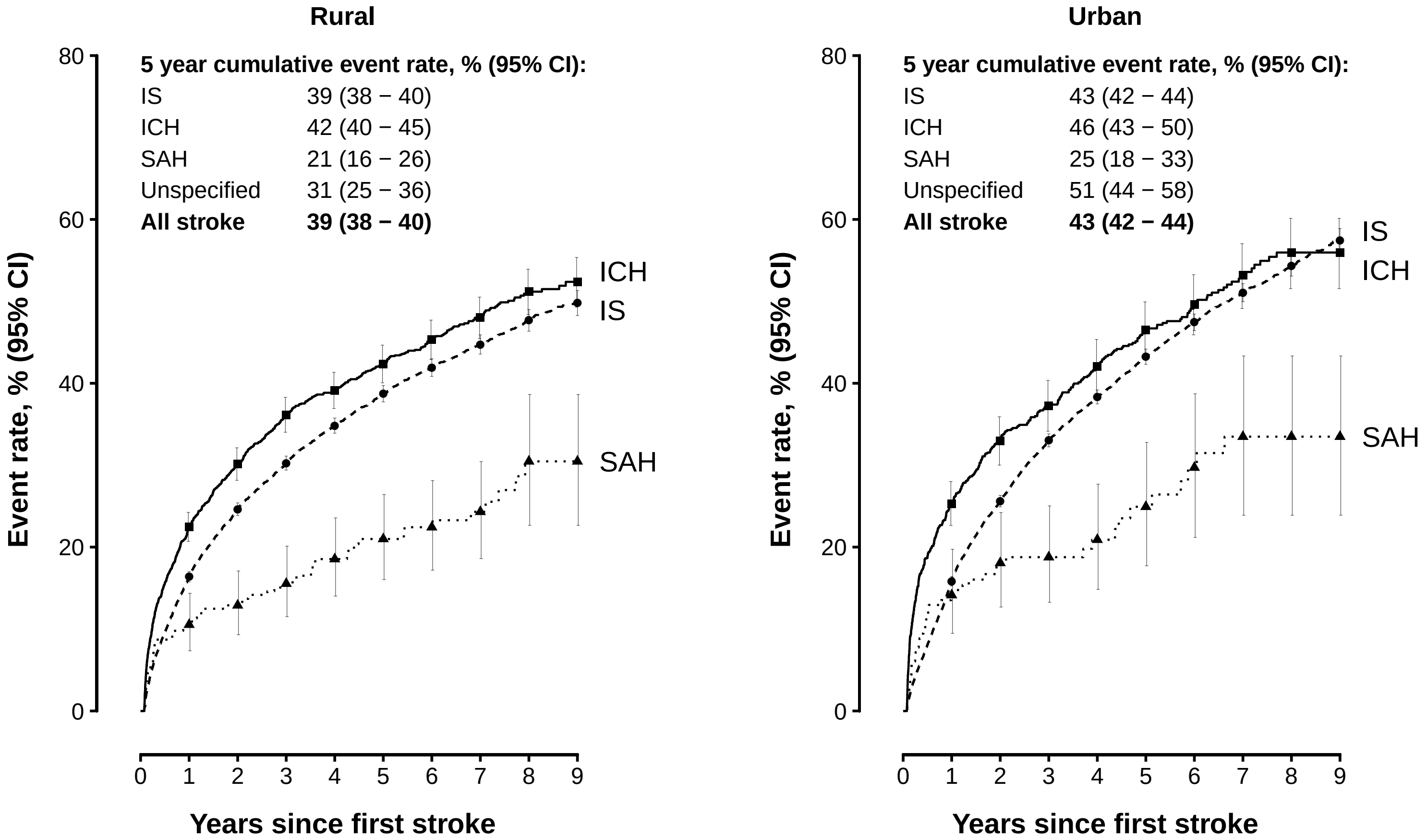

Rural: Numbers at risk	0	5	9	
	14,313	2,729	272	IS
	2,216	593	65	ICH
	301	111	14	SAH

Urban: Numbers at risk	0	5	9	
	16,919	3,158	250	IS
	1,046	213	21	ICH
	182	47	13	SAH

Web Figure 3: Estimated cumulative all−cause mortality rate from 28 days after first stroke of different types by (a) sex and (b) area

(a) Sex

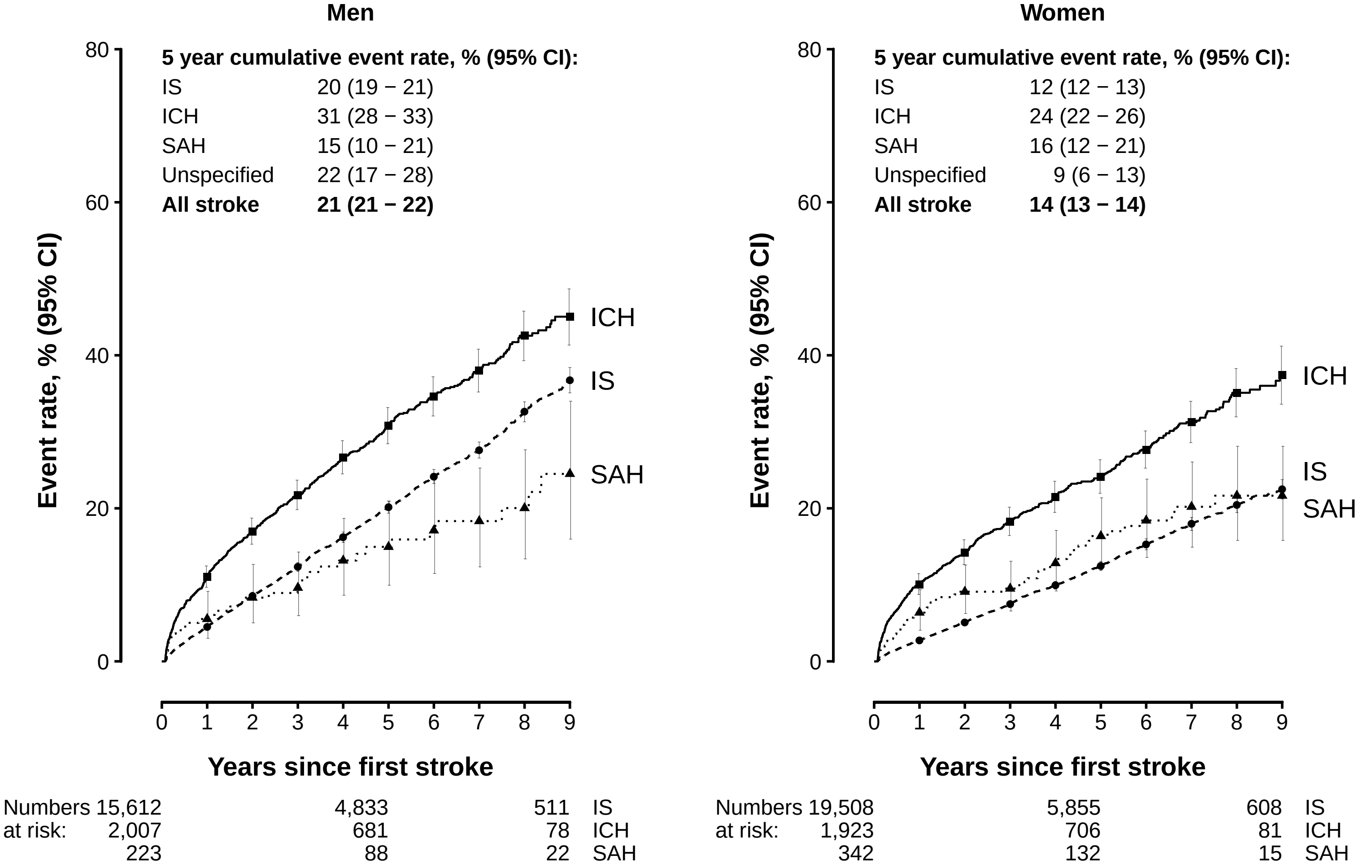

(b) Area

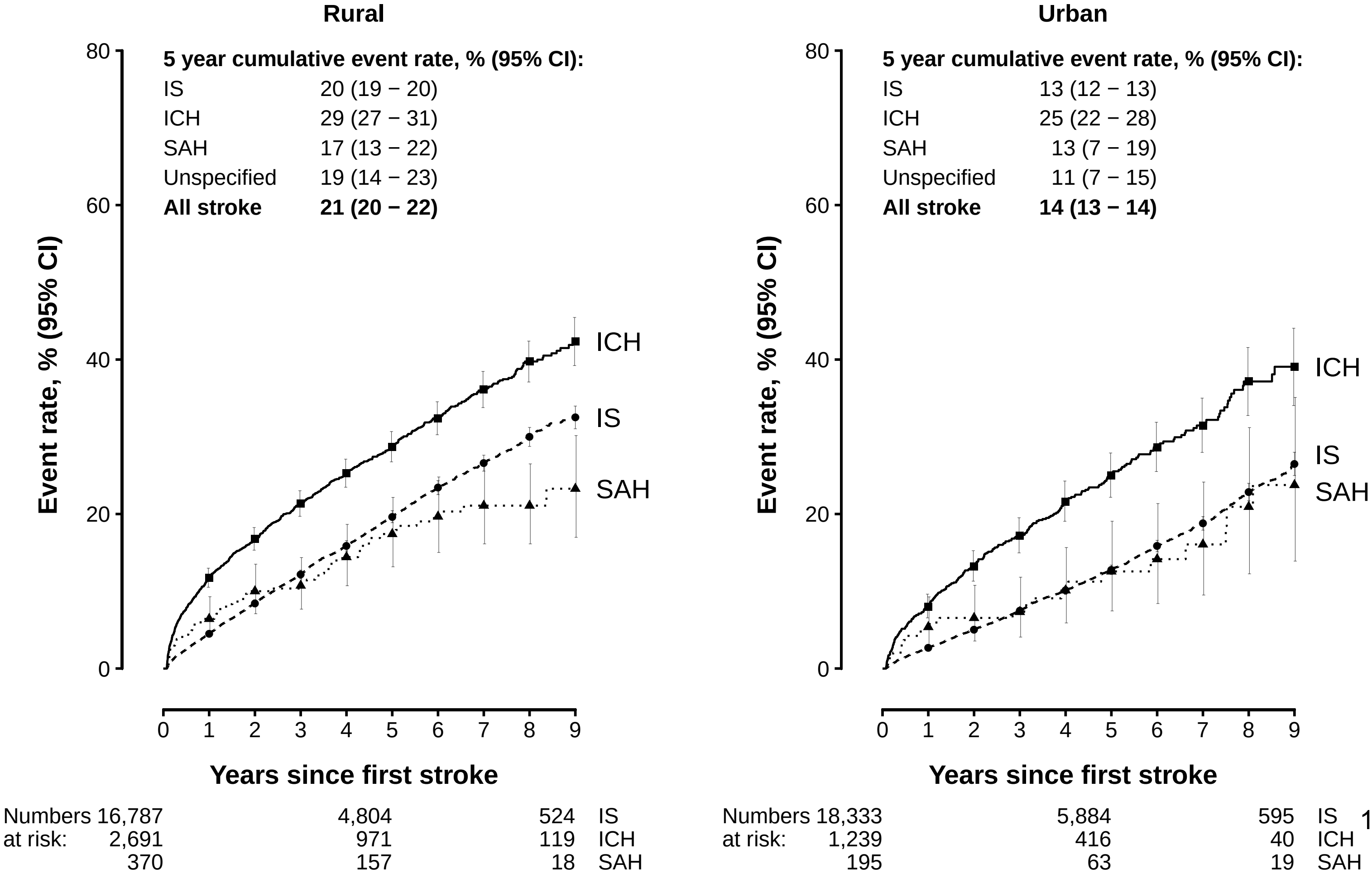

Web Figure 4: Estimated cumulative event rate of MI and other cardiac diseases from 28 days after first stroke of different types

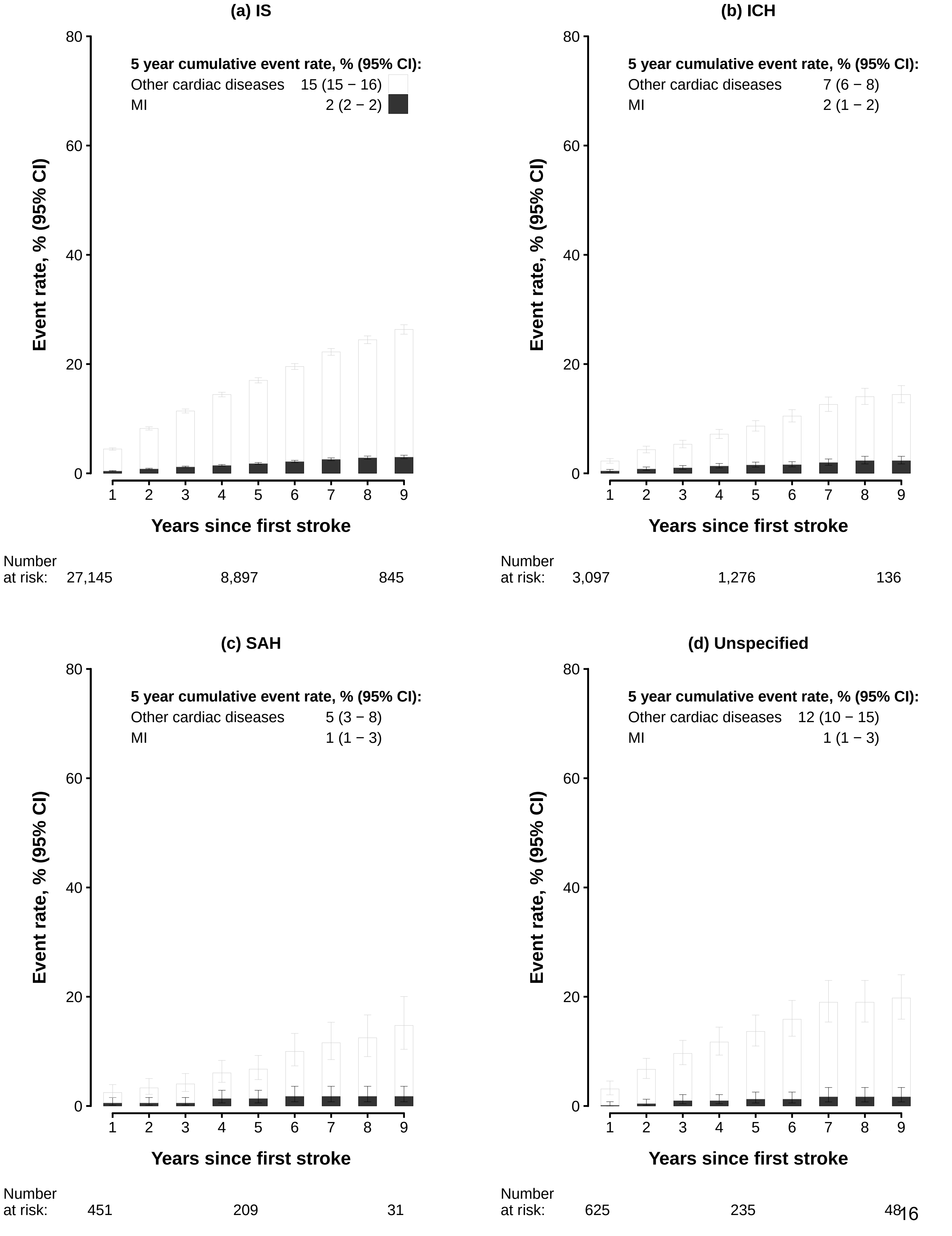

Supplement: Supplementary appendix [file mmc1.pdf]
